# High performance III-V photoelectrodes for solar water splitting via synergistically tailored structure and stoichiometry

**DOI:** 10.1038/s41467-019-11351-1

**Published:** 2019-07-29

**Authors:** Haneol Lim, James L. Young, John F. Geisz, Daniel J. Friedman, Todd G. Deutsch, Jongseung Yoon

**Affiliations:** 1 Mork Family Department of Chemical Engineering and Materials Science, University of Southern California, Los Angeles, CA 90089 USA; 2 National Renewable Energy Laboratory, Golden, CO 80401 USA; 3 Ming Hsieh Department of Electrical Engineering, University of Southern California, Los Angeles, CA 90089 USA

**Keywords:** Photocatalysis, Nanoscale materials, Devices for energy harvesting

## Abstract

Catalytic interface of semiconductor photoelectrodes is critical for high-performance photoelectrochemical solar water splitting because of its multiple roles in light absorption, electrocatalysis, and corrosion protection. Nevertheless, simultaneously optimizing each of these processes represents a materials conundrum owing to conflicting requirements of materials attributes at the electrode surface. Here we show an approach that can circumvent these challenges by collaboratively exploiting corrosion-resistant surface stoichiometry and structurally-tailored reactive interface. Nanoporous, density-graded surface of ‘black’ gallium indium phosphide (GaInP_2_), when combined with ammonium-sulfide-based surface passivation, effectively reduces reflection and surface recombination of photogenerated carriers for high efficiency photocatalysis in the hydrogen evolution half-reaction, but also augments electrochemical durability with lifetime over 124 h via strongly suppressed kinetics of corrosion. Such synergistic control of stoichiometry and structure at the reactive interface provides a practical pathway to concurrently enhance efficiency and durability of semiconductor photoelectrodes without solely relying on the development of new protective materials.

## Abstract (web summary)

Photoelectrochemical water splitting presents an integrated means storing sunlight into fuels, yet high optical losses and corrosion limit device performance. Here, authors boost absorption and durability in gallium-indium phosphide photocathodes via a sulfurized and nanostructured protection layer.

## Introduction

High-efficiency, high-durability materials for photoelectrochemical (PEC) solar water splitting have been the subject of intense research efforts over the past decades owing to their significant potential to economically produce a pollution-free energy carrier, hydrogen, as well as address the inherent intermittency of solar electricity^1–9^. Among various materials systems considered, a host of superior materials properties including a direct bandgap, appropriate band-edge energetics, as well as ability to monolithically form multiple solid-state junctions with an ideal bandgap combination for absorbing sunlight render III–V compound semiconductors such as gallium arsenide (GaAs) exceptionally attractive for photocatalytic electrodes in solar-driven photoelectrochemical water splitting^4,5,10–12^. Nonetheless, practical utilization of these materials in solar water splitting has been severely hampered largely due to their inherent thermodynamic instability against corrosion in a wide range of pH under relevant electrochemical potentials of water electrolysis reactions^13–16^. Developing robust protective materials for semiconductor photoelectrodes, however, represents a materials conundrum owing to sophisticated and often conflicting requirements of materials attributes at the reactive interface, where processes in optical absorption, charge transfer, electrocatalysis, as well as protection from corrosion must be simultaneously considered and optimized^13,16–19^.

Gallium indium phosphide (Ga_0.51_In_0.49_P, referred to as GaInP_2_), a ternary alloy lattice-matched to GaAs, is an indispensable materials component for realizing a ultrahigh (>20%) solar-to-hydrogen efficiency due to its near-ideal bandgap energy (~1.8 eV) for the large-bandgap material in multijunction photoelectrodes^4,5,12^. In such tandem systems of solar water splitting, the GaInP_2_-based top junction is directly exposed to a liquid electrolyte, thereby responsible for not only driving one of the water-splitting half reactions (i.e., hydrogen or oxygen evolution) but also serving as the first optical interface for receiving sunlight. Minimized optical losses and electrochemical durability in corrosive electrolytes are therefore two of the most important materials attributes desired for GaInP_2_ photoelectrodes. While Fresnel reflection at the semiconductor/water interface instantly loses a large fraction (~20–25%) of incident solar illumination^5,11^, dielectric thin films (e.g., Si_3_N_4_) frequently employed as an antireflective coating (ARC) in photovoltaic devices are not suitable for PEC photoelectrodes because of their inherent instabilities in highly acidic or alkaline electrolytes, poor charge transfer characteristics, and/or the low or lack of catalytic activities in water splitting reactions. In this regard, approaches that can reduce front-surface reflection while enhancing catalytic efficiency and corrosion resistance have been highly sought after as they can realize the full potential of GaInP_2_ and, in a broader context, III–V-based photoelectrodes in solar water splitting^10,20–23^. Here, we report a strategy that can address these challenges without solely relying on additionally deposited protective materials. The presented scheme takes advantage of a synergistic combination of density-graded nanoporous materials interface with sulfur-based stoichiometric control to simultaneously achieve enhanced light absorption and catalytic efficiency, as well as suppressed kinetics of corrosion. In the following, systematic studies of optical, morphological, compositional, and electrochemical properties, together with numerical optical modeling based on finite-difference time-domain method, provide quantitative description of underlying scientific principles in the reported system based on GaInP_2_, a key material that can make an immediate impact to the development of ultrahigh efficiency solar-driven water splitting systems.

## Results

### Fabrication of surface-tailored GaInP_2_ photocathodes

**Fig. 1:**
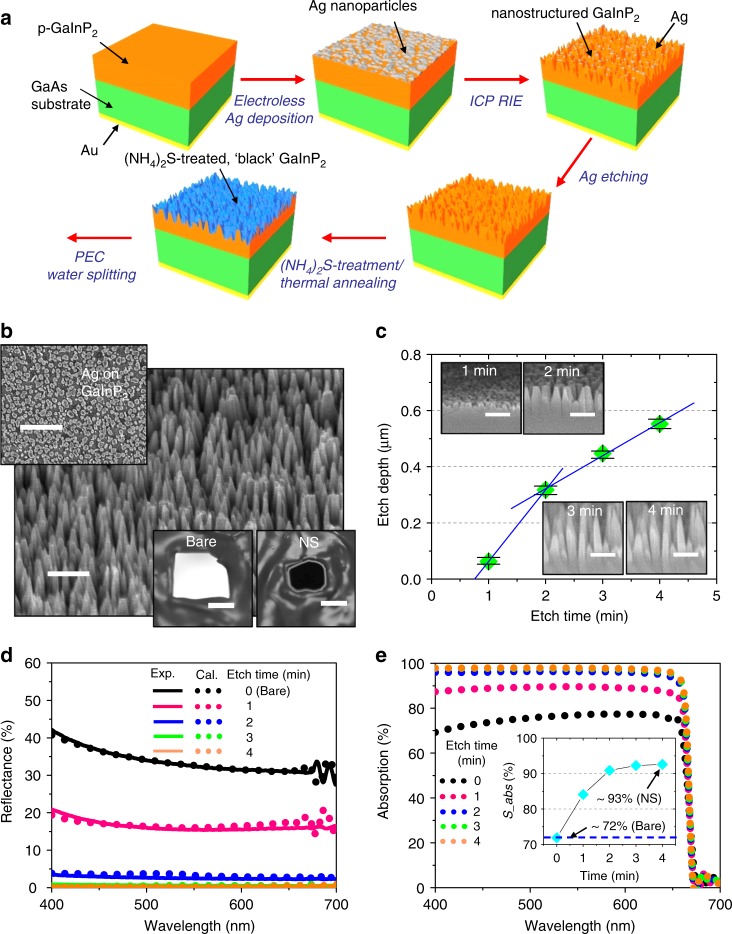
Schematic illustration, morphological and optical properties of surface-tailored GaInP_2_ photocathodes. **a** Schematic illustration of fabrication procedures for surface-tailored black GaInP_2_ photocathodes. **b** Tilt-view scanning electron microscope (SEM) image of nanostructured p-type GaInP_2_ photocathodes after the dry etching and before the (NH_4_)_2_S-treatment (scale bar: 500 nm). The top inset shows a SEM image of electrolessly deposited silver nanoparticles on GaInP_2_ as a mask for dry etching (scale bar: 1 μm). The bottom inset shows photographic images of fully functional bare (labeled as “Bare”) and nanostructured (labeled as “NS”) GaInP_2_ photocathodes mounted on a slide glass with epoxy encapsulation (scale bar: 5 mm), where the black surface of nanostructured GaInP_2_ is evidently shown in contrast to the shiny surface of bare GaInP_2_. **c** Etching depth of the nanostructured GaInP_2_ (yet without (NH_4_)_2_S-treatment) measured from cross-sectional SEM images (inset) at etching times of 1, 2, 3, and 4 min (scale bar: 300 nm). Error bars represent the range of values obtained from three separate measurements (*n* = 3). **d** Corresponding total (i.e., specular and diffuse) reflectance spectra of nanostructured GaInP_2_ measured on spectrophotometer equipped with an integrating sphere at an incidence angle of 8°. Calculated (dotted line) reflectance spectra obtained from FDTD-based numerical optical modeling matched well with the experimental (solid line) spectra. **e** Calculated absorption spectra of nanostructured GaInP_2_ in water using the numerical model established in **d**. The inset shows corresponding integrated solar flux absorption (*S_abs*)

As an active material for photocathodes in solar water electrolysis, p-type (Zn-doped, 2 × 10^17^ cm^−3^) GaInP_2_ with a thickness of 2.5 μm was grown on a (100) GaAs substrate by an atmospheric pressure metal organic vapor phase epitaxy (MOVPE)^5,24^. Figure 1a schematically illustrates fabrication procedures for surface-tailored GaInP_2_ photocathodes. The process begins with the oxide-removal of as-grown p-type GaInP_2_ in dilute NH_4_OH and chromium etchant, followed by dipping into an aqueous solution of silver nitrate (AgNO_3_) and hydrofluoric acid (HF). The silver cations in the solution are electrolessly reduced to form silver nanoparticles that can serve as a hard mask in successive dry etching of GaInP_2_, where the size and density of silver nanoparticles can be readily controlled by adjusting the concentration of precursors and/or plating time^25,26^. Subsequently, inductively coupled plasma reactive ion etching (ICP RIE) was performed using a gas mixture of BCl_3_/N_2_ to form cone-shaped nanopillars of GaInP_2_, followed by the removal of residual silver by a wet chemical etchant. After the formation of nanoporous morphology, chemical passivation of nanostructured surface was performed (Supplementary Fig. 1). The surface of ‘black’ GaInP_2_ was soaked in an aqueous solution of ammonium sulfide ((NH_4_)_2_S), followed by thermal annealing in air to incorporate corrosion-resistant surface stoichiometry. In the subsequent discussion, this two-step process consisting of (NH_4_)_2_S-passivation and thermal annealing is referred to as (NH_4_)_2_S-treatment unless additional specifications are provided. Figure 1b shows a tilt-view scanning electron microscope (SEM) image of the representative GaInP_2_ nanostructure after the dry etching (yet without (NH_4_)_2_S-treatment). The diameter of nanopillars gradually decreased from the bottom to the tip, thereby creating a graded index of refraction to suppress front-surface reflection^26,27^. The surface-tailored GaInP_2_ was then electrically connected with a copper wire on the metal contact at the backside of the sample and encapsulated by thermally cured epoxy to produce fully functional photocathodes ready for driving the hydrogen evolution half-reaction in solar water splitting^28^.

### Optical properties of black GaInP_2_ photocathodes

Efficient coupling of sunlight into the semiconductor photoelectrode is one of the key advantages for the reported black GaInP_2_^23,26^. The average heights of GaInP_2_ nanostructure measured from the cross-sectional SEM images (Fig. 1c) were approximately ~60, ~320, ~450, and ~550 nm for etching times of 1, 2, 3, and 4 min, respectively. Overall, the height of nanopillars increased with the time of dry etching, while the rate of increase diminished after 2 min under the present experimental conditions. Figure 1d shows the corresponding total (i.e., sum of diffuse and specular) reflectance of nanostructured GaInP_2_ at near-normal incidence (θ = 8°) in air. The tapered nanopillars strongly suppressed the front-surface reflectance over a broad wavelength range owing to the improved impedance matching arising from a gradually varying refractive index^26,29,30^. The reflectance at 500 nm decreased from ~33% for bare GaInP_2_ to less than ~1% for nanostructured (3-min-etched) samples (Supplementary Fig. 2). The measured reflectance (solid line) quantitatively matched with the calculated spectra (dotted line) obtained from 3D full-wave numerical optical modeling based on finite-difference time-domain method (FDTD, Lumerical^TM^) (Supplementary Fig. 3)^29,31^. Using the established numerical model, we also projected the absorption enhancement of nanostructured GaInP_2_ in the electrolyte (i.e., water) (Fig. 1e). The inset shows integrated solar flux absorption (*S*_abs_) weighted over a simulated AM1.5 G solar illumination calculated by

1 $$S_{{\mathrm{abs}}}({\mathrm{\% }}) = \frac{{\mathop {\smallint }\nolimits_{400{\mathrm{nm}}}^{678{\mathrm{nm}}} \frac{\lambda }{{hc}}A(\lambda )I_{1.5{\mathrm{G}}}(\lambda ){\mathrm{d}}\lambda }}{{\mathop {\smallint }\nolimits_{400{\mathrm{nm}}}^{678{\mathrm{nm}}} \frac{\lambda }{{hc}}I_{1.5{\mathrm{G}}}(\lambda ){\mathrm{d}}\lambda }} \times 100$$

where *h*, *c*, *A*(λ), and *I*_1.5G_ (λ) are Planck’s constant, the speed of light, calculated absorption, and the standard solar irradiance (AM 1.5 G; ASTM G-173), respectively^11,29^. The *S*_abs_ of black GaInP_2_ in water is ~92% (for 3-min-etched sample), which is considerably higher than the maximum absorption (~72%) of bare (i.e., without nanopillars) GaInP_2_ in water and can be directly translated to the enhanced electrode efficiency. These density-graded morphologies and reflectance spectra of dry-etched GaInP_2_ were also preserved after the (NH_4_)_2_S-treatment (Supplementary Fig. 4).

### PEC performance of black GaInP_2_ photocathodes in the HER

**Fig. 2:**
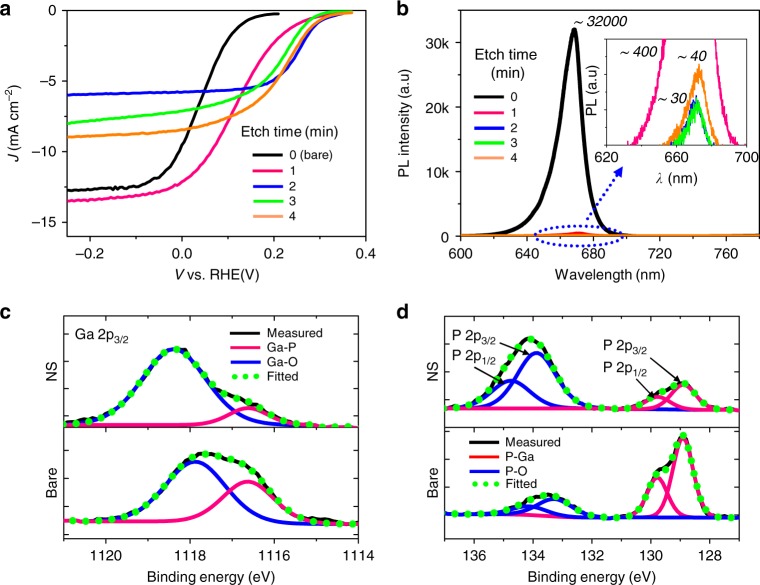
Photoelectrochemical performance of nanostructured GaInP_2_ photocathodes for the HER. **a** Representative *J–E* curves of bare and nanostructured GaInP_2_ photocathodes driving the hydrogen evolution reaction (HER), measured under simulated AM1.5 G solar illumination (1000 W/m^2^). All samples were measured without (NH_4_)_2_S-treatment. **b** Corresponding steady-state photoluminescence (PL) spectra of bare and nanostructured GaInP_2_ photocathodes. **c** XPS spectra of Ga 2p_3/2_ for bare and nanostructured GaInP_2_. The measured spectra (black line) matched quantitatively with fitted spectra (green dotted line) composed of deconvoluted Ga–O (blue line) and Ga–P (red line) peaks. **d** XPS spectra of P 2p_1/2_ and 2p_3/2_ for bare and nanostructured GaInP_2_. The fitted spectra were deconvoluted to resolve P–Ga (red line) and P–O (blue line) peaks

The PEC characteristics of black GaInP_2_ photocathodes in the hydrogen evolution reaction (HER) were studied in a three-electrode configuration under simulated AM1.5G solar illumination (1000 W m^−2^), where Pt and Ag/AgCl were used as counter and reference electrodes, respectively, with aqueous sulfuric acid (0.5 M H_2_SO_4_) as an electrolyte (Supplementary Fig. 5)^11^. All samples were measured without (NH_4_)_2_S-treatment. Figure 2a shows current density (*J*)-potential (*E*) curves of the nanostructured GaInP_2_ photocathodes prepared at various etching times, obtained from a linear sweep voltammetry from −0.5 to 0.4 V (vs. reversible hydrogen electrode (RHE)), where the data from the first scan were plotted (Supplementary Fig. 6). The efficiency (*η*_cathode_) of GaInP_2_ photocathodes for the HER was calculated by,

2 $$\eta _{{\mathrm{cathode}}}({\mathrm{\% }}) = \frac{{J_{{\mathrm{max}}} \cdot (E_{{\mathrm{max}}} - E(H^ + /H_2))}}{{P_{{\mathrm{in}}}}} \times 100,$$

where *J*_max_ and *E*_max_ are the current density and electrode potential at a maximum power point, *E(H*^*+*^*/H*_*2*_*)* is the thermodynamic HER potential, and *P*_in_ is the power density of simulated AM1.5G solar illumination^11,32^. While this diagnostic efficiency at three-electrode configuration does not fully capture the solar-to-hydrogen (STH) efficiency of overall water splitting reactions, it is employed here as a metric to compare the electrode performance quantitatively^32^. As summarized in Supplementary Table 1, the onset potential (*V*_onset_) of all nanostructured GaInP_2_ photocathodes anodically (i.e., positively along the *x*-axis) shifted compared to the bare GaInP_2_ because of the reduction of local current density associated with the enlarged surface area and corresponding decrease of over-potential^10,23^. Although a dark current is also increased with the enlarged surface area, the kinetic advantage of a reduced local current density dominates in the trade-off of diminished voltage of a nanostructured surface with a large dark current. Consequently, both fill factor and efficiency greatly improved in nanostructured samples. On the other hand, the saturated current density (*J*_sat_) slightly increased for 1-min-etched sample compared to the bare GaInP_2_ owing to the suppressed reflection loss but became smaller at longer etching times. This observation indicates a large degree of surface recombination of photogenerated carriers caused by plasma-induced crystalline defects and oxidation at the nanostructured surface, as supported by the severe attenuation of steady-state photoluminescence (PL) with the dry-etched GaInP_2_ (Fig. 2b, Supplementary Fig. 7). X-ray photoelectron spectroscopy (XPS) studies also support this analysis. In Fig. 2c, the Ga 2p_5/2_ peaks with binding energies of 1118.0 and 1116.7 eV observed from bare GaInP_2_ correspond to 3+ oxidation states for Ga_2_O_3_ and GaInP_2_, respectively. Notably, the integrated area of Ga–O peak, reflecting the relative amount of Ga–O bonding, substantially increased after the dry etching, suggesting the incorporation of oxygen atoms at the etched surface of GaInP_2_ and generation of defect states within the bandgap that can act as centers for non-radiative carrier recombination^33,34^. With similar origins, the relative amount of P–O (In–O) peaks increased over P–Ga (In–P) peaks after the formation of nanopillars by dry etching (Fig. 2d, Supplementary Fig. 8).

### Effect of (NH_4_)_2_S-treatment on PEC performance

**Fig. 3:**
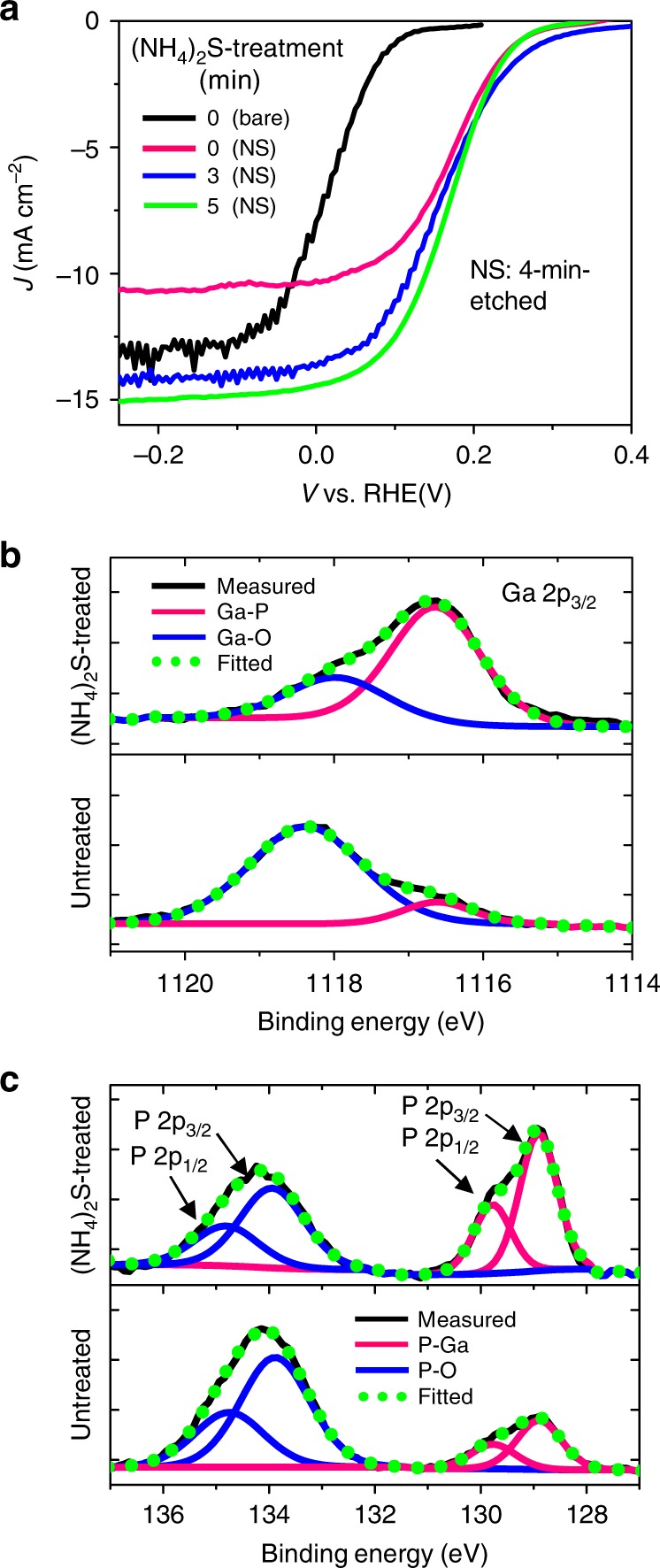
Photoelectrochemical performance of nanostructured and (NH_4_)_2_S-treated GaInP_2_ photocathodes for the HER. **a** Representative *J-E* curves of bare and nanostructured GaInP_2_ photocathodes for the HER after the (NH_4_)_2_S-treatment of 3 and 5 min, measured under simulated AM1.5 G solar illumination (1000 W/m^2^). XPS spectra of **b** Ga 2p_3/2_ and **c** P 2p_1/2_ and 2P_3/2_ for nanostructured GaInP_2_ before and after the (NH_4_)_2_S-treatment (15 min)

To take a full advantage of black GaInP_2_ in light absorption, it is therefore essential to address the issue of increased carrier recombination associated with etching-induced surface oxidation but also to protect the nanoporous morphology from corrosion. To this end, we soaked the nanostructured GaInP_2_ in a dilute solution of ammonium sulfide ((NH_4_)_2_S), followed by thermal annealing in air (250 °C for 1 h, Supplementary Fig. 1)^35,36^. Figure 3a shows *J–E* curves of 4-min etched GaInP_2_ photocathodes before and after the (NH_4_)_2_S-treatment, plotted with the data for bare GaInP_2_ as a reference. For samples before the sulfur passivation, the saturated current density (~10.9 mA cm^−2^) of nanostructured GaInP_2_ was smaller than that (~13.3 mA cm^−2^) of bare GaInP_2_ owing to the above-described non-radiative carrier recombination. By contrast, the *J*_*sat*_ of the nanostructured GaInP_2_, after the (NH_4_)_2_S-treatment for 3 and 5 min, recovered to ~14.4 and ~15.2 mA cm^−2^, respectively, resulting in a large improvement of diagnostic efficiency (*η*_cathode_) by over 100% (relative) compared to the untreated samples (Supplementary Table 2). Such large enhancement of *J*_sat_ was also accompanied by the partial recovery of PL intensities (Supplementary Fig. 9), as well as the reduced areas of Ga–O-related, P–O-related, and In–O-related peaks in XPS spectra (Fig. 3b, c, Supplementary Fig. 10), suggesting that the substitution of oxygen atoms by sulfur at the surface of nanostructured GaInP_2_ and corresponding decrease of oxide-related defect states effectively lowered the extent of surface recombination and thus restored the efficiency of charge transfer at the catalytic interface^35,37^. Along with the (NH_4_)_2_S-treatment, the performance of surface-tailored GaInP_2_ can be further enhanced by additionally depositing materials of high catalytic activity (i.e., co-catalyst) on the electrode surface such as noble metals or molecular catalysts. In the present study, a thin (~10–30 nm, Supplementary Fig. 11) layer of amorphous molybdenum disulfide (MoS_2_) was photochemically deposited on the nanostructured and (NH_4_)_2_S-treated GaInP_2_ as a HER co-catalyst (Supplementary Figs. 12 and 13). As expected, the MoS_2_ co-catalyst markedly improved the catalytic performance of surface-tailored GaInP_2_ in the HER, with a large enhancement in both onset potential and fill factor, resulting in the substantial increase of efficiency by ~20 times compared to the bare electrode (Supplementary Table 3).

### Electrochemical stability of surface-tailored GaInP_2_

**Fig. 4:**
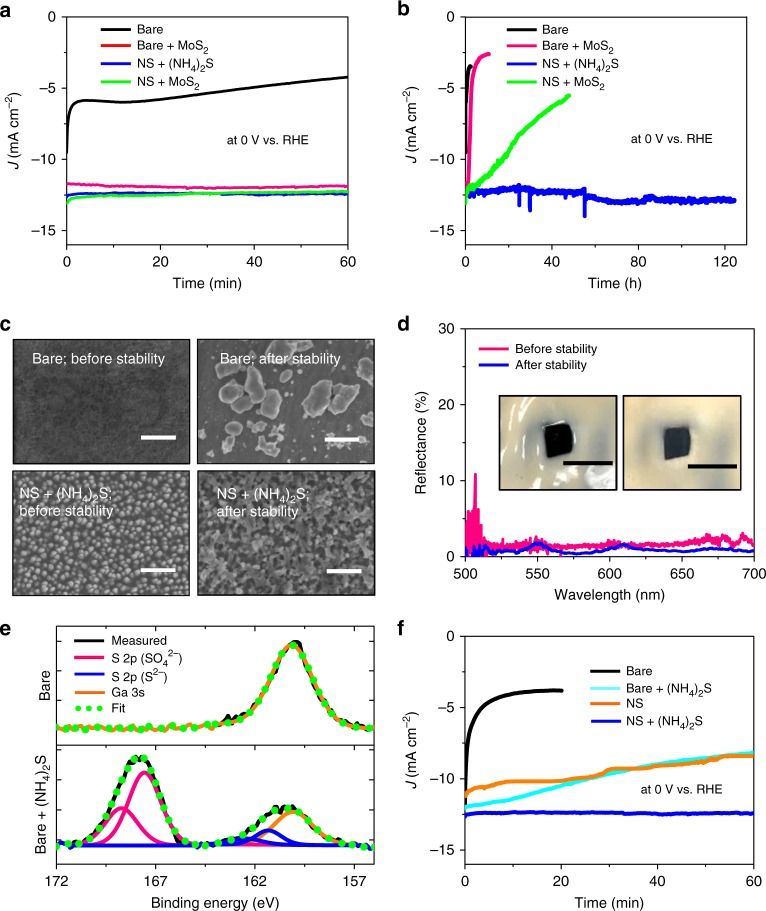
Electrochemical durability of surface-tailored GaInP_2_ photocathodes performing the HER under bias. Current density–time (*J–t*) plots of GaInP_2_ photocathodes in an acidic electrolyte (0.5 M H_2_SO_4_) for **a** short-term and **b** long-term measurements, at various materials configurations including bare GaInP_2_ (black data), bare GaInP_2_ deposited with MoS_2_ (yet without (NH_4_)_2_S-treatment, red data), nanostructured GaInP_2_ with (NH_4_)_2_S-treatment (yet without MoS_2_-deposition, blue data), and nanostructured GaInP_2_ with MoS_2_ (yet without (NH_4_)_2_S treatment, green data), measured at an electrode potential of 0 V (vs. RHE) under simulated AM1.5 G solar illumination. Dry etching and (NH_4_)_2_S-treatment were performed for 4 min and 15 min, respectively. **c** Top-view SEM images of bare and nanostructured/(NH_4_)_2_S-treated GaInP_2_ (scale bar: 500 nm) before and after the stability test in **b** (i.e., ~1 h for bare, ~124 h for NS). **d** Reflectance spectra of nanostructured and (NH_4_)_2_S-treated GaInP_2_ photocathodes before and after the stability test in **b**. Insets show corresponding photographic images of samples (scale bar: 5 mm). **e** XPS spectra of bare GaInP_2_ before and after the (NH_4_)_2_S-treatment. **f** *J-t* plots of bare and nanostructured GaInP_2_ photocathodes with and without (NH_4_)_2_S-treatment, obtained under the same measurement condition as in **a**

Long-term preservation of intrinsic materials properties and catalytic performance of semiconductor photoelectrodes is one of the most critical requirements for their practical application in solar water splitting. Nevertheless, III–V compound semiconductors including GaInP_2_ suffer from intrinsic thermodynamic instability and corrode rapidly in a wide range of pH under the potentials of water splitting reactions, thereby leading to the fast degradation of electrode functionality with an impractically short lifetime^6,14,15,38^. In this regard, the reported surface-tailoring strategy provides a potential route to strongly enhance the durability of III–V photoelectrodes. Figure 4a, b shows the current density of GaInP_2_ photocathodes as a function of time for various materials configurations including bare GaInP_2_ (i.e., with unetched and untreated surface), bare GaInP_2_ deposited with MoS_2_ co-catalyst (yet without (NH_4_)_2_S-treatment), nanostructured GaInP_2_ with (NH_4_)_2_S treatment (yet without MoS_2_-deposition), and nanostructured GaInP_2_ with MoS_2_-deposition (yet without (NH_4_)_2_S-treatment), measured at an electrode potential of 0 V (vs. RHE) in an acidic electrolyte (0.5 M H_2_SO_4_) under simulated AM1.5 G solar illumination. Dry etching and (NH_4_)_2_S-treatment were performed for 4 min and 15 min, respectively. In short-term measurements (i.e., up to ~60 min, Fig. 4a), all tested samples exhibited nearly constant current densities except the bare GaInP_2_, where the rapid degradation of *J* with bare electrodes (black data) is attributed to the cathodic shift of the *JE* curve arising from the formation of surface oxide in water, which gradually plateaued due to the self-limiting nature of wet oxidation^28^. The bare electrode deposited with MoS_2_ (red data) showed comparatively stable performance because of the temporary prevention of surface oxidation by the MoS_2_ layer. It is noteworthy that the nanostructured GaInP_2_ after the (NH_4_)_2_S-treatment (blue data) remained stable even without the aid of additional protective materials. The extraordinary durability was pronounced more evidently in long-term measurements as depicted in Fig.4b. The current density of nanostructured GaInP_2_ with (NH_4_)_2_S-treatment (blue data) was maintained nearly undiminished (*ΔJ* < ~2%) for over ~124 hours, where the measurement was terminated without observing the degradation of electrode performance. It is also notable that the onset potential continuously improved during the stability test (Supplementary Figs. 14–16), which might be attributed to several factors including the activation of catalytic sites of sulfurized GaInP_2_, as well as enhanced photovoltage and charge transfer efficiency, all occurring with the removal of oxides and/or carbon-containing species that are unstable in the HER (Supplementary Note 1, Supplementary Figs. 17 and 18). By contrast, the bare electrodes both with (red data) and without MoS_2_ (black data) rapidly degraded fast in the early stage (<3 h) of measurement. In case of the nanostructured GaInP_2_ with MoS_2_ but without (NH_4_)_2_S-treatment (green data), significant degradation was still noted owing to the delamination or dissolution of MoS_2_ during the HER. Consistent with these observations, the density-graded surface morphology of nanostructured and (NH_4_)_2_S-treated GaInP_2_ remained nearly intact after the chronoamperometry study (Fig. 4b), as evidenced by the SEM images (Fig. 4c), as well as the preservation of black appearance and low reflectance (Fig. 4d). For bare electrodes, by contrast, significant degrees of corrosion already proceeded just after the ~2.5-h measurement, where particles with sizes ranging from a few microns to tens of nanometers appeared on the electrode surface as reported in the previous literature^39,40^.

To further elucidate the synergistic contributions of (NH_4_)_2_S-treatment and nanoporous surface morphology to the extraordinary improvement of corrosion-resistance, we examined the evolution of surface atomic composition in bare GaInP_2_ by XPS before and after the (NH_4_)_2_S-treatment (Fig. 4e), as well as with and without thermal annealing during the (NH_4_)_2_S-treatment (Supplementary Fig. 19). It is noteworthy that the (NH_4_)_2_S-treated bare GaInP_2_ that exhibited stable performance has peaks corresponding to sulfate (SO_4_^2−^) group (the lower spectra in Fig. 4e), which was introduced during the thermal annealing in the (NH_4_)_2_S-treatment^8,9^ (Supplementary Fig. 19). Such sulfate (SO_4_^2−^)-related and sulfide (S^2-^)-related signals are, however, completely missing in untreated bare GaInP_2_ (the upper spectra in Fig. 4e) that degraded rapidly. It is therefore concluded that the sulfate group on the sulfurized surface of GaInP_2_ played a key role in the strongly enhanced durability. On the other hand, the (NH_4_)_2_S-treated bare GaInP_2_ (i.e., without surface nanostructure, cyan data in Fig. 4f) did not exhibit a long-term durability comparable to the (NH_4_)_2_S-treated and nanostructured GaInP_2_ (blue data in Fig. 4f), while it was still more stable than the untreated bare electrode (black data in Fig. 4f), suggesting that the synergistic interplay between the nanostructured morphology to enlarge the surface area and limit the progression of corroded region, and the incorporation of sulfide/sulfate group to confer the improved corrosion resistance is collectively responsible for suppressing the kinetics of corrosion and enabling significantly longer lifetime observed in this study compared to previous works (Supplementary Table 4).

## Discussion

The change of surface morphology observed after the stability test with nanostructured, (NH_4_)_2_S-treated GaInP_2_ might be attributed to the desorption and re-adsorption of Ga, In, P, or S atoms at the catalytic interface, through either faradaic or non-faradaic processes, with the latter independent of corrosion reactions that would be parasitic to the photocurrent. Faradaic efficiencies of H_2_ measured from bare GaInP_2_ without and with (NH_4_)_2_S-treatment are ~80 and ~91% (Supplementary Table 5, Supplementary Fig. 20), respectively, possibly due to the contribution of corrosion to the photocurrent as observed in Fig. 4f. As expected, the faradaic efficiency of (NH_4_)_2_-treated GaInP_2_ is higher than the untreated GaInP_2_ owing to combined effects of surface passivation and suppressed corrosion on the sulfurized reactive interface. Nevertheless, the nanoporous morphology was maintained in ways that produce similar levels of reflectance and light absorption, and thus the photocurrent to those obtained before the stability test. Given that the degradation of (NH_4_)_2_S-treated bare GaInP_2_ has also accompanied the gradual decrease of sulfate group on the electrode surface as evidenced by XPS spectra (Supplementary Fig. 21), we postulate that the rate of electrochemical dissolution of sulfate group is substantially lowered by the nanoporous morphology owing to the reduced rate of local charge transfer associated with the enlarged surface area. While the nanostructured GaInP_2_ without (NH_4_)_2_S-treatment (orange data in Fig.4f) exhibited improved durability compared to the untreated bare electrode, it also steadily degraded at the rate much higher than the nanostructured and (NH_4_)_2_S-treated GaInP_2_, reiterating the importance of collaborative contributions from nanoporous surface morphology and corrosion-resistant surface stoichiometry.

In summary, we demonstrated an approach that can simultaneously enhance the light absorption, catalytic efficiency, and durability of GaInP_2_ photocathodes in the HER of solar water splitting by collaboratively exploiting corrosion-resistant surface stoichiometry and structurally tailored reactive interface. The sulfur treatment has been demonstrated effective for passivating the surface states and suppressing the surface recombination in a wide range of semiconductor materials^37,41,42^. We therefore expect our approach capitalizing the synergistic effect of surface nanostructure and corrosion-resistant surface stoichiometry would be broadly applicable to various semiconductor photoelectrodes (e.g., III–V, III-N) and electrochemical reactions (e.g., oxygen evolution reaction (OER), CO_2_ reduction) that can benefit from simultaneously enhanced light absorption, catalytic efficiency, and corrosion resistance, all without solely relying on the development of new protective materials, thereby offering practical pathways towards high efficiency, high durability PEC solar water splitting.

## Methods

### Fabrication of surface-tailored GaInP_2_ photocathodes

A 2.5 μm-thick p-type GaInP_2_ (Zn-doped, 2 × 10^17^ cm^−3^) was epitaxially grown on a GaAs (100) substrate miscut 2° towards the (110) at 700 °C by an atmospheric pressure metal organic vapor phase epitaxy (MOVPE). The as-received GaInP_2_ ep-wafer was cleaned with acetone, isopropyl alcohol (IPA), and deionized (DI) water, followed by the removal of native oxides in a dilute NH_4_OH solution (NH_4_OH (29%, EMD):DI water = 1:10, by volume, 2 min) and perchloric acid solution (CR-7, KMG, 30 s). Silver nanoparticles were electrolessly deposited on GaInP_2_ in an aqueous solution of silver nitrite (AgNO_3_, 10 mM) and hydrofluoric acid (HF, 5 M). Nanostructured GaInP_2_ photocathodes were formed by inductively coupled plasma reactive ion etching (ICP-RIE, STS) (BCl_3_:N_2_ (1.5:9 in sccm), 5 mTorr, 100 W/500 W, 100 °C) using silver nanoparticles as an etch mask. Subsequently, the residual silver was removed by a wet chemical etchant (NH_4_OH:H_2_O_2_:DI water = 1:1:1, by volume). After the (NH_4_)_2_S-treatment and/or MoS_2_-deposition as described in detail subsequently, a copper wire was connected using a silver paste on the back of the wafer, followed by the encapsulation of side walls using thermally cured epoxy (Loctite® 9462) to produce fully functional photocathodes.

### PEC measurements

All PEC measurements were performed in an aqueous solution (0.5 M, pH: ~0.3–0.35) of sulfuric acid (H_2_SO_4_, EMD Chemicals, ACS grade, 95–98%) under simulated AM 1.5 G standard solar illumination (1000 W/m^2^) on a full-spectrum solar simulator (94042 A, Oriel) at ambient temperature (20 °C). The one-sun intensity of solar simulator was calibrated using a certified reference cell (91150 V, Newport). The electrolyte solution was purged with N_2_ for 15 min before each PEC measurement. Linear sweep voltammetry data were collected by a potentiostat (Reference 600, Gamry) under a three-electrode configuration with Ag/AgCl (3 M NaCl, RE-5B, Bioanalytical Systems) and platinum (MW-1032, Bioanalytical Systems) as reference and counter electrodes, respectively, where the potential of the working electrode was scanned from –0.8 V to 0.04 V vs. Ag/AgCl (−0.57 V to 0.27 V vs. RHE) for bare GaInP_2_, from −0.8 V to 0.14 V vs. Ag/AgCl (−0.57 to ~0.37 V vs. RHE) for nanostructured GaInP_2_, and from −0.8 V to ~0.4 V vs. Ag/AgCl (−0.57 V to ~0.63 V vs. RHE) for MoS_2_-deposited GaInP_2_, respectively, at a scan rate of 20 mV/s in a step size of 5 mV. Note that the potential sweep was done from negative to positive potential to avoid overestimation of electrode performance (Supplementary Fig. 5). A small amount of surfactant (Triton X-100, SPI Supplies) was added to the electrolyte to facilitate the release of generated hydrogen bubbles from the electrode surface. For the conversion of electrode potential from Ag/AgCl to the reversible hydrogen electrode (RHE), a linear sweep voltammetry scan was performed using a platinum electrode (MF-2013, Bioanalytical Systems) as a cathode to experimentally determine the onset potential of hydrogen evolution. The current density (*J*) was evaluated based on the measured area of illuminated electrode surface (Supplementary Note 2). For Figs. 2a, 3a, 4a, b, f, representative data from two or three batches of experiments were reported.

### (NH_4_)_2_S-treatment

To tailor the surface atomic composition of GaInP_2_, a pre-heated (NH_4_)_2_S solution (~61 °C, ~0.77 M, prepared by adding 34 mL of DI water or isopropanol to 10 mL of as-received (NH_4_)_2_S solution (Macron, 20.0–24.0 wt% in H_2_O)) was cast onto the surface of bare or nanostructured GaInP_2_ placed on a hot plate (~85 °C) for 3–5 min (for samples in Fig. 3a). The treatment time for samples in Fig. 3b, c, and 4 was 15 min. Subsequently, samples were dried under N_2_, followed by thermal annealing at 250 °C in air for 1 h.

### MoS_2_-deposition

For MoS_2_-deposition, the GaInP_2_ was immersed in an aqueous solution of (NH_4_)_2_MoS_4_ (1 mM, Sigma Aldrich) and 0.5 M of Na_2_SO_4_ buffer (pH 6.6) under white light (LED-6WD, AmScope) illumination (~20 mW/cm^2^) for 5 min at an open-circuit condition, followed by rinsing with DI water and drying under N_2_. After the photochemical deposition, the sample was thermally annealed at 250 °C under N_2_ atmosphere for 1 h. Alternatively, MoS_2_ was also electrochemically deposited by scanning 8–10 times of potential cycles between −0.4 and +0.15 V vs. Ag/AgCl (i.e., one cycle: −0.4 V → +0.15 V → −0.4 V) using the same regents as in photochemical deposition under a dark condition.

### Reflectance measurement

Reflectance spectra of GaInP_2_ photocathodes (Fig. 1d) were recorded using UV-Vis-NIR spectroscopy (Lamda 950, Perkin-Elmer) at near-normal incidence (*θ* = 8°) in air, measured on a spectrophotometer equipped with an integrating sphere using a Spectralon® as a 100% reflectance standard. For the data in Fig. 4d, reflectance spectra were recorded using a home-made optical set-up consisting of a white light source (HL-2000, Ocean Optics) and a fiber-optic spectrometer (Flame-T-VIS-NIR, Ocean Optics). The source light was collimated by an achromatic doublet lens (*f* = 19 mm, N.A. = 0.42) and then focused on the cell region (beam diameter = ~50 μm) through an objective lens (×20, N.A. = 0.4). The reflected light was collected by the same objective lens and guided to the spectrometer through a multimode fiber. A silver mirror deposited on fused silica (PF10-03-P01, Thorlabs) was used as a 100% calibration standard.

### Photoluminescence measurement

Photoluminescence (PL) spectra of bare and nanostructured GaInP_2_ samples were measured using a Raman microscope (XploRA^TM^, HORIBA Jobin Yvon Inc.) with ×100 objective lens (NA: 0.90), where a 532-nm laser was focused on the sample surface with a beam diameter of ~1 μm.

### X-ray photoelectron spectroscopy (XPS)

XPS was performed on a Kratos Axis Ultra DLD, where photoelectrons were generated by monochromatic Al Kα X-ray at 1486.7 eV at a base pressure of 4 × 10^−8^ torr. Binding energies were calibrated by C 1 s peak at 284.8 eV. Linear-least-squares fitting of XPS spectra was performed using CasaXPS software (Casa Software Ltd.) using convolution of Gaussian (70%) and Lorentzian (30%) line-shapes. The peak positions and relative intensity ratios are nearly identical between bare and nanostructured samples under the present experimental conditions (Supplementary Fig. 22).

### Atomic force microscopy (AFM) and ellipsometry

The thickness of MoS_2_ was measured by tapping-mode atomic force microscope (AFM, Dimensional 3100, Digital Instrument). Refractive index (*n*) and extinction coefficient (*k*) of GaInP_2_ were measured by spectroscopic ellipsometry (VASE® Ellipsometer, J.A.Woollam).

### Numerical optical modeling

Reflectance and absorption spectra of nanostructured GaInP_2_ photocathodes were numerically modeled by finite-difference time-domain method (FDTD). To produce a model nanostructured surface, top-view SEM micrographs (~1.2 × 0.9 μm^2^) of the dry-etched GaInP_2_ at various etching times were imported to a 3D modeling software (Rhinoceros®), where lateral profiles of nanopillars were adjusted to closely match with those observed experimentally. The created nanostructured surface was further imported to the FDTD software (FDTD Solutions, Lumerical^TM^). For calculation at normal incidence, a 3D simulation volume was confined with periodic boundary conditions for the *x*-direction and *y*-direction, and a perfectly matched layers (PML) boundary condition for the *z*-direction, where a continuous plane wave that has a broad Gaussian frequency spectrum (270–750 THz or 400–1100 nm) was assumed as a light source.

### Faradaic efficiency measurement

Hydrogen and oxygen gases were collected volumetrically by a Hoffman-type apparatus from bare GaInP_2_ photocathodes with and without (NH_4_)_2_S-treatment. The photocathodes were operated at 0 V vs. RHE using Pt and Hg/Hg_2_SO_4_ (MSE) as counter and reference electrodes, respectively, in 0.5 M sulfuric acid illuminated by a tungsten-halogen lamp with water filter calibrated to one Sun intensity using a GaInP_2_ reference cell. Faradaic efficiency was calculated with the following equation:

3 $$\begin{array}{l}\eta _{H_2} = \frac{{{\mathrm{Collected}}\,{\mathrm{gas}}\,{\mathrm{quantity}}({\mathrm{mol}})}}{{{\mathrm{Expected}}\,{\mathrm{gas}}\,{\mathrm{quantity}}({\mathrm{mol}})}}\\ {\hskip46pt}= \frac{{\left( {\frac{{P_{H_2}V}}{{RT}}} \right)}}{{\left( {{\mathrm{Charges}}\,{\mathrm{passed}}} \right) \times \left( {\frac{{{\mathrm{mol}}\,e^ - }}{{96485C}}} \right) \times \left( {\frac{{1\,{\mathrm{mol}}\,H_2}}{{2\,{\mathrm{mol}}\,e^ - }}} \right)}},\end{array}$$

where $P_{H_2}$ is the pressure of the evolved hydrogen gas, *V* is the volume, *R* is the gas constant (62363 mL·torr·K^−1^·mol^−1^) and *T* is temperature (292.59 K). More detailed procedures of faradaic efficiency measurements appear elsewhere^5^.

### Reporting summary

Further information on research design is available in the Nature Research Reporting Summary linked to this article.

## Supplementary information

Solar Cells Reporting Summary

Supplementary Information

## Data Availability

The data that support the plots within this paper and other findings of this study are available from the corresponding authors upon reasonable request.
