# Supplementary material for: High performance III-V photoelectrodes for solar water splitting via synergistically tailored structure and stoichiometry
Source: Nat Commun. 2019 Jul 29;10:3388. doi: 10.1038/s41467-019-11351-1 (PMC6662753; doi:10.1038/s41467-019-11351-1)
Supplement: Supplementary file 1 — Solar Cells Reporting Summary [file 41467_2019_11351_MOESM1_ESM.pdf]

nature research

Corresponding author(s): Jongseung Yoon

Solar Cells Reporting Summary

Nature Research wishes to improve the reproducibility of the work that we publish. This form is intended for publication with all accepted papers reporting the characterization of photovoltaic devices and provides structure for consistency and transparency in reporting. Some list items might not apply to an individual manuscript, but all fields must be completed for clarity.

For further information on Nature Research policies, including our data availability policy, see Authors & Referees.

Experimental design

Please check: are the following details reported in the manuscript?

1. Dimensions

Area of the tested solar cells
☒ Yes ☐ No
This information is available in Supplementary Note.

Method used to determine the device area
☒ Yes ☐ No
This information is available in Supplementary Note.

2. Current-voltage characterization

Current density-voltage (J-V) plots in both forward and backward direction
☐ Yes ☒ No
In the photocatalysis with III-V semiconductor photocathode, a single sweep JE curve from negative to positive potentials has been the common practice, since the scan from the positive to negative potentials can overestimate performance.

Voltage scan conditions
For instance: scan direction, speed, dwell times
☒ Yes ☐ No
This information is available in Methods section.

Test environment
For instance: characterization temperature, in air or in glove box
☒ Yes ☐ No
This information is available in Methods section.

Protocol for preconditioning of the device before its characterization
☐ Yes ☒ No
Preconditioning is not necessary for III-V solar cells and photoelectrodes.

Stability of the J-V characteristic
Verified with time evolution of the maximum power point or with the photocurrent at maximum power point; see ref. 7 for details.
☒ Yes ☐ No
JE characteristics after PEC stability tests were provided in Supplementary Figs. 14 and 15.

3. Hysteresis or any other unusual behaviour

Description of the unusual behaviour observed during the characterization
☐ Yes ☒ No
No hysteresis or unusual behavior of solar cell was observed.

Related experimental data
☐ Yes ☒ No
No hysteresis or unusual behavior of solar cell was observed.

4. Efficiency

External quantum efficiency (EQE) or incident photons to current efficiency (IPCE)
☐ Yes ☒ No
EQE or IPCE measurements were not performed in this study as they are not required to support the claims of this study.

A comparison between the integrated response under the standard reference spectrum and the response measure under the simulator
☐ Yes ☒ No
EQE or IPCE measurements were not performed in this study.

For tandem solar cells, the bias illumination and bias voltage used for each subcell
☐ Yes ☒ No
Tandem solar cells were not studied.

5. Calibration

Light source and reference cell or sensor used for the characterization
☒ Yes ☐ No
This information is available in Methods section.

Confirmation that the reference cell was calibrated and certified
☒ Yes ☐ No
This information is available in Methods section.

November 2017

Calculation of spectral mismatch between the reference cell and the devices under test

☐ Yes ☒ No

Calculation of spectral mismatch was not performed as it is not required to support the claims of this study.

6. Mask/aperture

Size of the mask/aperture used during testing

☐ Yes ☒ No

Mask or aperture was not used during testing as the device performance in this study is not affected by them.

Variation of the measured short-circuit current density with the mask/aperture area

☐ Yes ☒ No

Measured short-circuit current density in this study is not affected by the mask/aperture area.

7. Performance certification

Identity of the independent certification laboratory that confirmed the photovoltaic performance

☐ Yes ☒ No

Performance certification was not carried out as it is not required to support the claims of this study.

A copy of any certificate(s)
Provide in Supplementary Information

☐ Yes ☒ No

Performance certification was not carried out.

8. Statistics

Number of solar cells tested

☒ Yes ☐ No

This information is available in Methods section.

Statistical analysis of the device performance

☐ Yes ☒ No

PEC measurements in Figs. 2a, 3a, 4a, 4b, 4f involve the comparison of electrode performance for multiple samples prepared by different processing conditions, where representative data from 2 or 3 sets of samples were reported for each figure. Statistical analysis among different sample batches is not required to support the conclusion of these comparison experiments.

9. Long-term stability analysis

Type of analysis, bias conditions and environmental conditions
For instance: illumination type, temperature, atmosphere humidity, encapsulation method, preconditioning temperature

☒ Yes ☐ No

Long-term stability analysis of photoelectrodes during the hydrogen evolution reaction was included in the main text (Figure 4).
