# Supplementary material for: High performance III-V photoelectrodes for solar water splitting via synergistically tailored structure and stoichiometry
Source: Nat Commun. 2019 Jul 29;10:3388. doi: 10.1038/s41467-019-11351-1 (PMC6662753; doi:10.1038/s41467-019-11351-1)
Supplement: Supplementary file 2 — Supplementary Information [file 41467_2019_11351_MOESM2_ESM.pdf]

Supplementary Information

High Performance III-V Photoelectrodes for Solar Water Splitting via Synergistically Tailored Structure and Stoichiometry

Haneol Lim[1], James L. Young[2], John F. Geisz[2], Daniel J. Friedman[2], Todd G. Deutsch[2], Jongseung Yoon*[1,3]

[1]Mork Family Department of Chemical Engineering and Materials Science, University of Southern California, Los Angeles, California 90089, USA.

[2]National Renewable Energy Laboratory, Golden, Colorado 80401, USA.

[3]Ming Hsieh Department of Electrical Engineering, University of Southern California, Los Angeles, California 90089, USA.

E-mail: js.yoon@usc.edu

Supplementary Figures

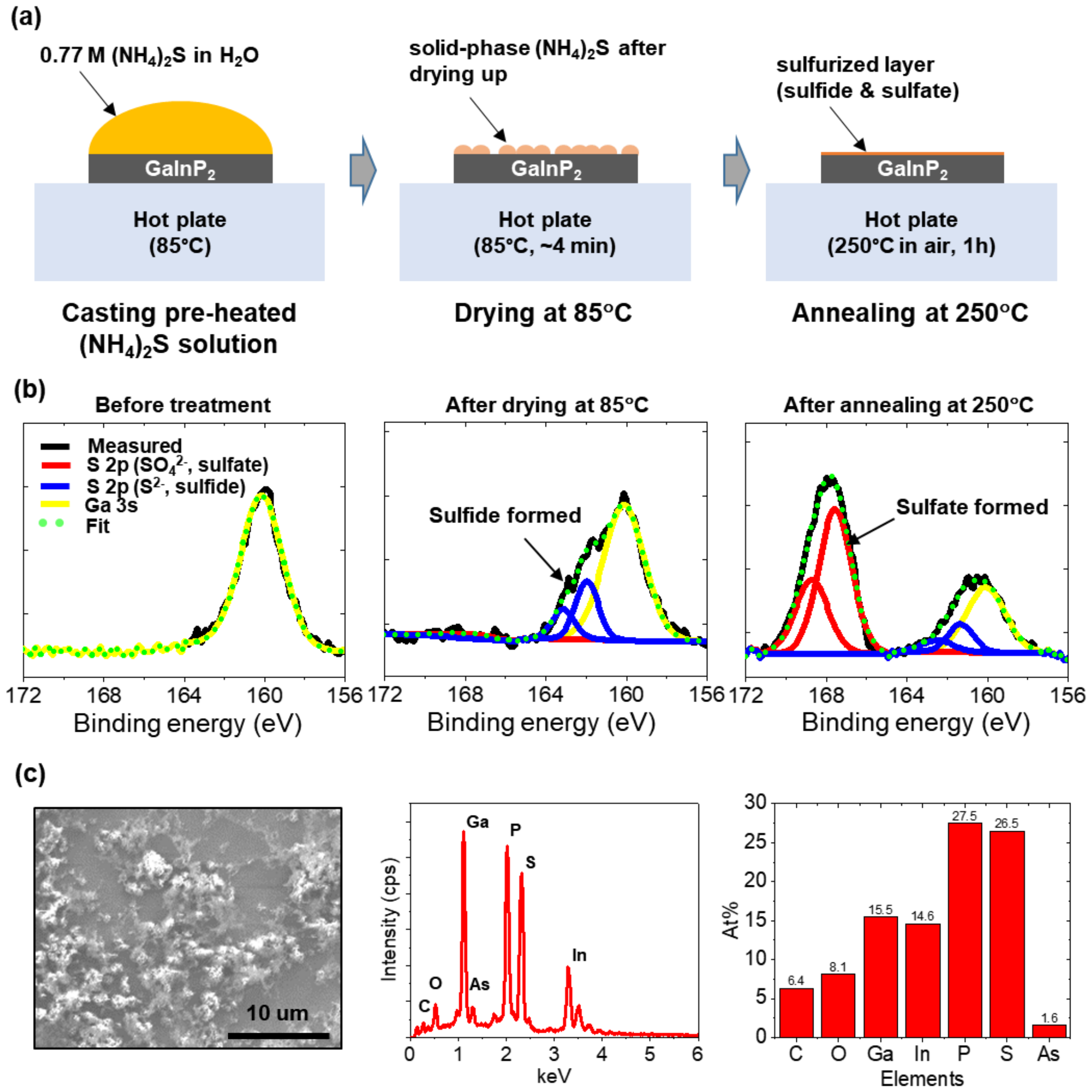

Supplementary Figure 1. Schematic illustration, XPS, SEM data of $(NH_4)_2S$-treatment

(a) Schematic illustration of two-step $(NH_4)_2S$ treatment. (b) XPS spectra of S 2p and Ga 3s before $(NH_4)_2S$-treatment (left), after drying at 85°C (center), and after heating at 250°C in air (right), supporting the formation of sulfide and sulfate species at each step. The second XPS spectra of S 2p were measured after washing the sample with DI water to remove a thick polysulfide layer shown on (c). (c) SEM image and energy dispersive spectroscopy (EDS) from the sample right after the casting at 85°C without washing in DI water, clearly showing that thick islands of $(NH_4)_2S$-based polysulfides and the formation of carbon-containing species after this first step of $(NH_4)_2S$-treatment.

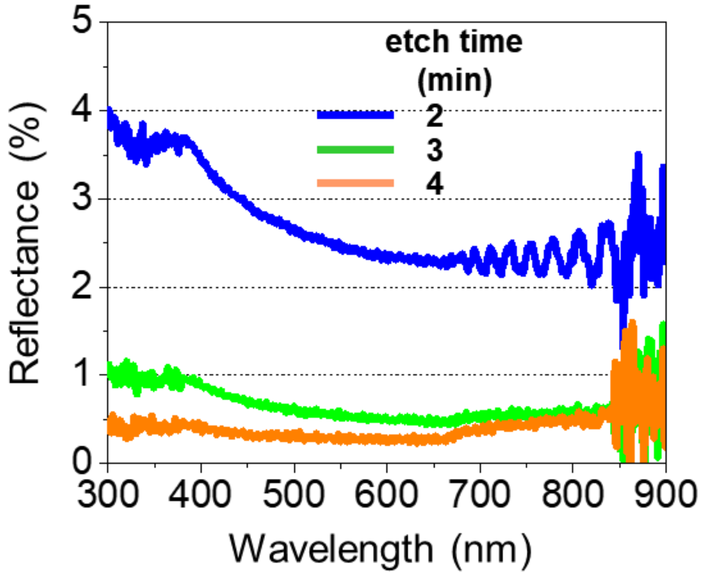

Supplementary Figure 2. Reflectance spectra of black $GaInP_2$

Zoomed-in, total reflectance spectra of Figure 2c, measured from nanostructured $GaInP_2$ (without $(NH_4)_2S$-treatment) at various etching times.

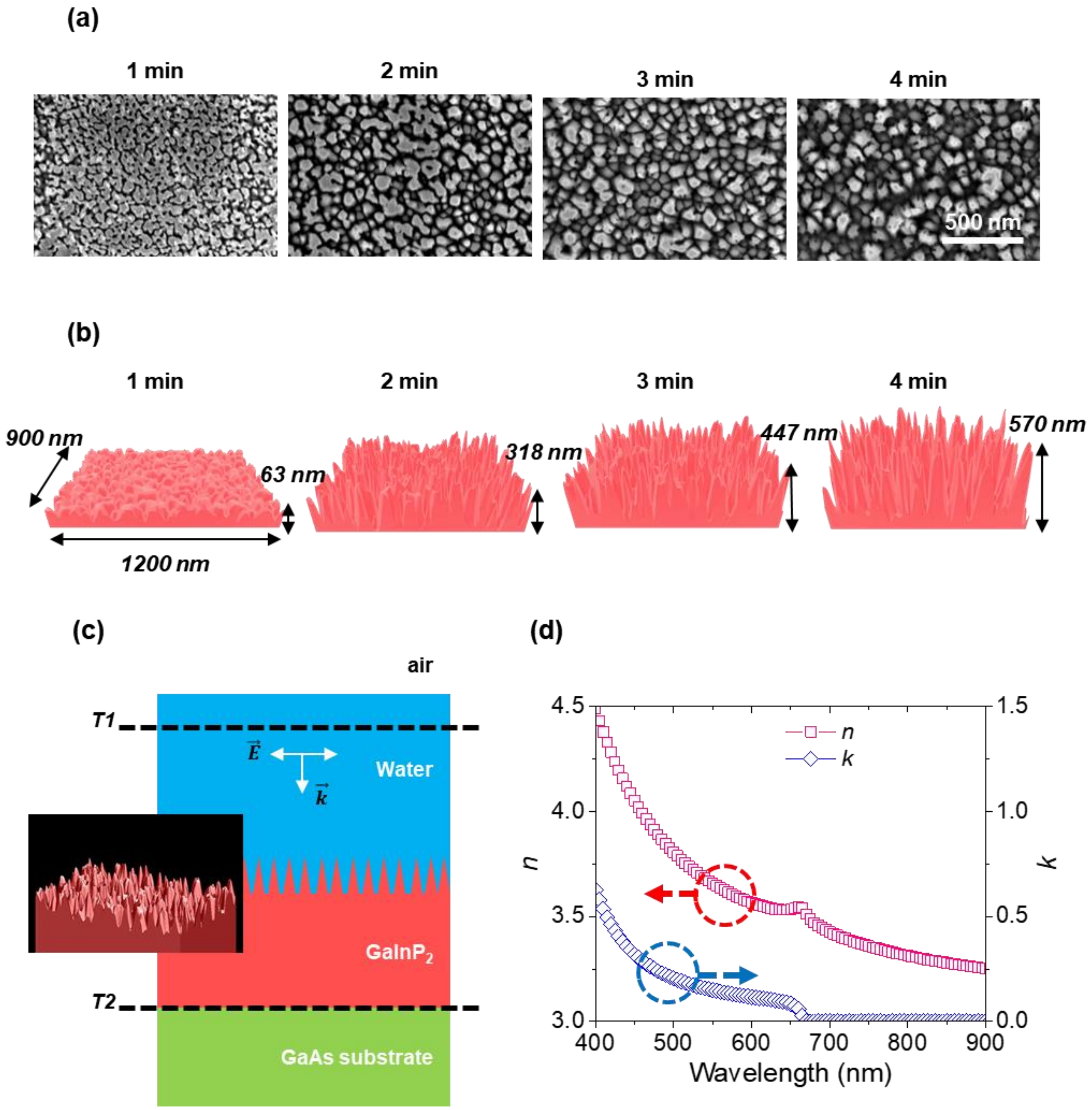

Supplementary Figure 3. Numerical optical modeling of black $GaInP_2$

(a) Top-view SEM images of nanostructured $GaInP_2$ (without $(NH_4)_2S$-treatment) at various etching times. (b) Tilt-view schematic illustration of constructed nanostructured surfaces by 3D modeling software (Rhinoceros®) using the SEM images in (a). (c) Cross-sectional schematic illustrations of nanostructured $GaInP_2$ for the FDTD calculation of reflectance and absorption spectra in water, where *T1* and *T2* indicate the positions of 'Transmission Monitors' in Lumerical™. The inset shows a nanostructured $GaInP_2$ implemented in Lumerical™. (d) Measured refractive index (n) and extinction coefficient (k) of $GaInP_2$ by spectroscopic ellipsometry, which were used in optical calculations.

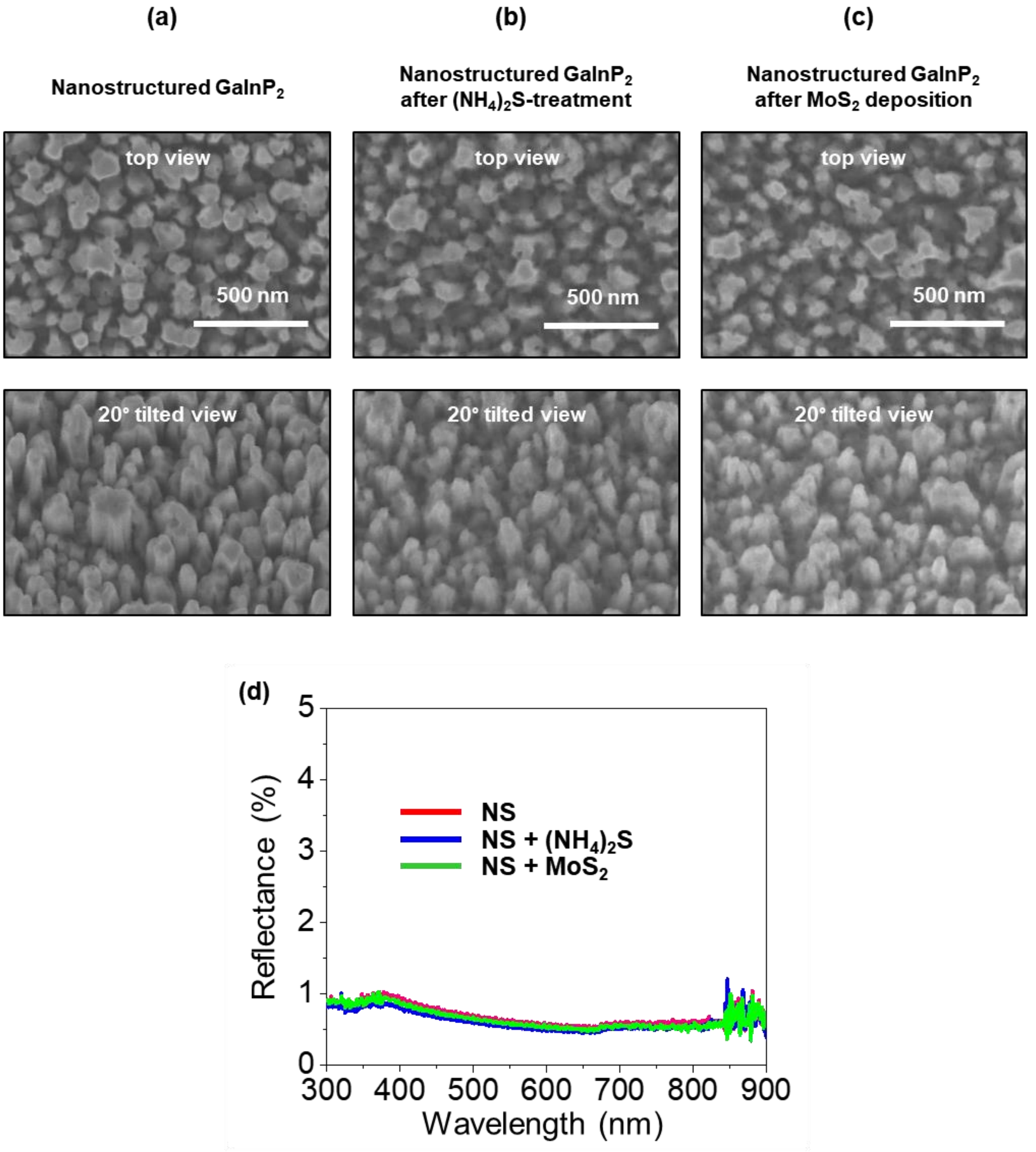

Supplementary Figure 4. Morphology of black $GaInP_2$ after MoS_2-deposition
SEM images of (a) nanostructured (4-min etching) $GaInP_2$ without $(NH_4)_2S$-treatment nor MoS_2-deposition, (b) nanostructured $GaInP_2$ after $(NH_4)_2S$-treatment (15 min, yet without MoS_2-deposition) and (c) nanostructured $GaInP_2$ after MoS_2-deposition (yet without $(NH_4)_2S$-treatment). All samples used in (a), (b), and (c) were prepared together using one piece of wafer up to the process of dry-etching, and they were cleaved into three pieces for each case. (d) Corresponding measured total reflectance spectra.

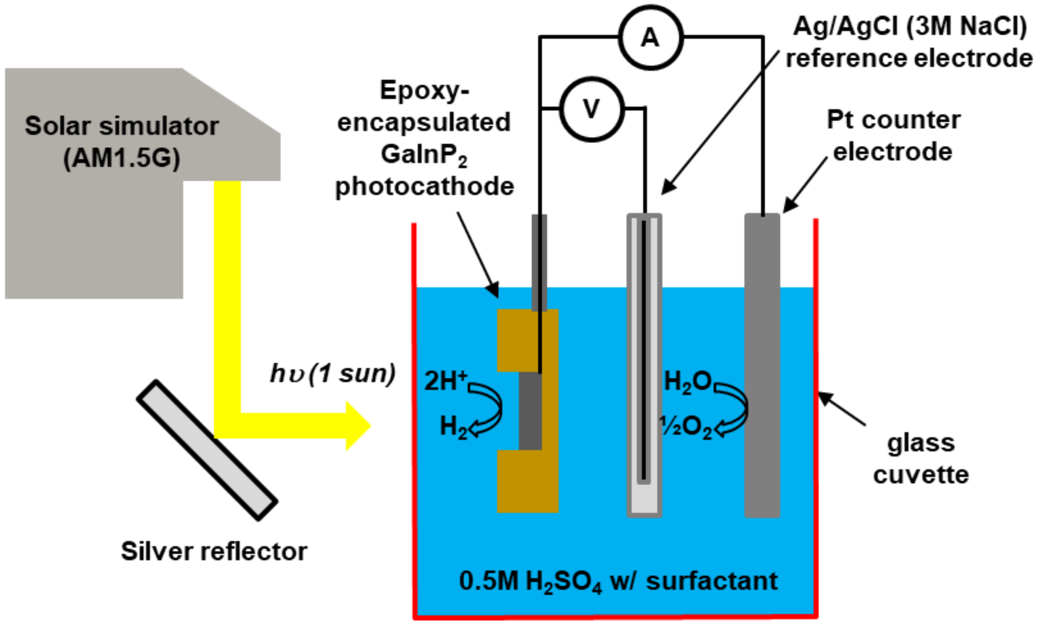

Supplementary Figure 5. Experimental setup for PEC measurements

A schematic illustration of PEC measurements in a three-electrode configuration with $GaInP_2$ photocathodes in solar water splitting.

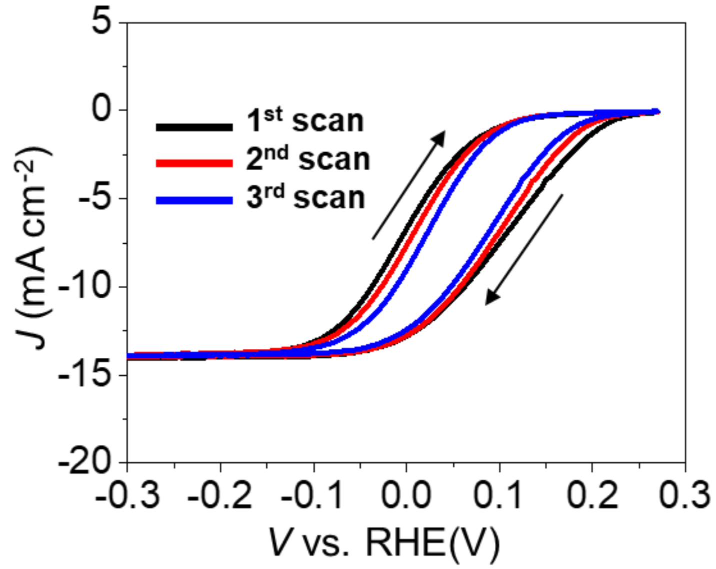

Supplementary Figure 6. Effect of scan direction in cyclic voltammetry
Cyclic voltammetry scans of bare $GaInP_2$ with no surface treatments measured in a three-electrode configuration under simulated AM1.5G solar illumination (1000 W/m^2), where Pt and Ag/AgCl were used as counter and reference electrodes, respectively, with aqueous sulfuric acid (0.5M H_2SO_4) as an electrolyte.

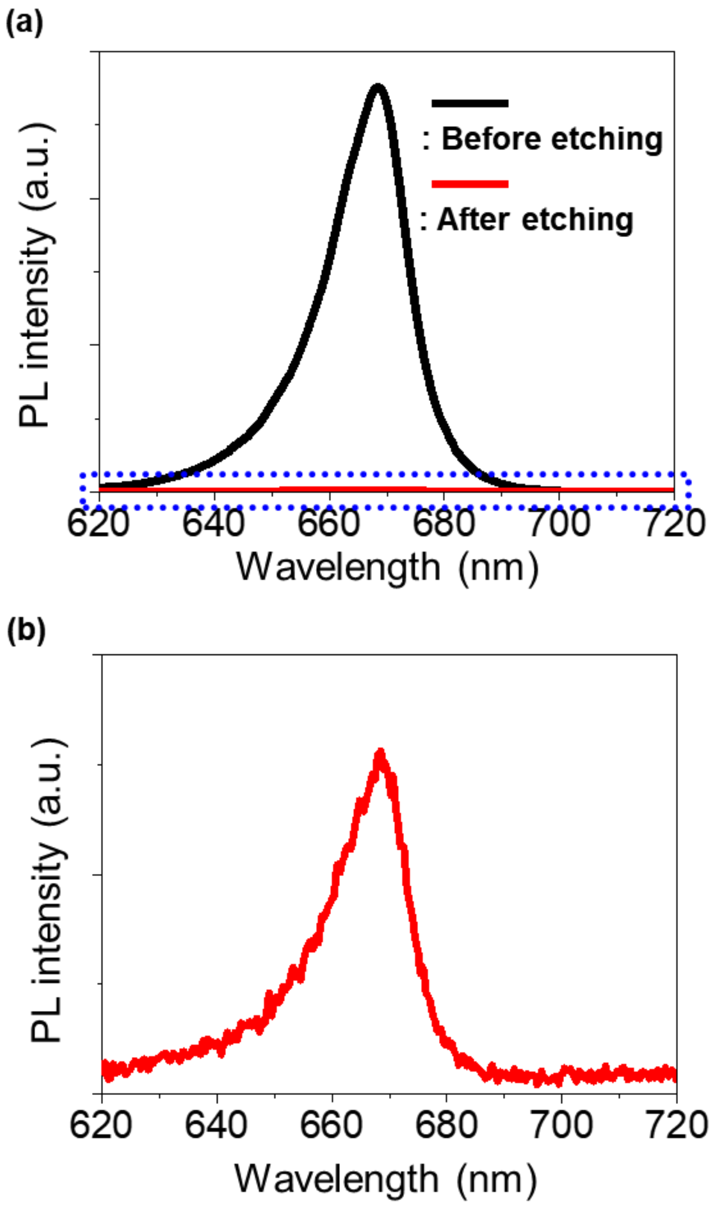

Supplementary Figure 7. Photoluminescence of bare and black $GaInP_2$

(a) Steady-state photoluminescence (PL) spectra of as-received bare $GaInP_2$ before and after the dry-etching (4 min) measured at room temperature. The etching was performed without silver nanoparticles using the same condition (BCl_3/N_2 (1.5/9.0 sccm), 100W/500W, 5 mTorr, 100°C) as for nanostructured $GaInP_2$, and (b) the same plot in the zoomed-in scale of y-axis (dotted region in (a)).

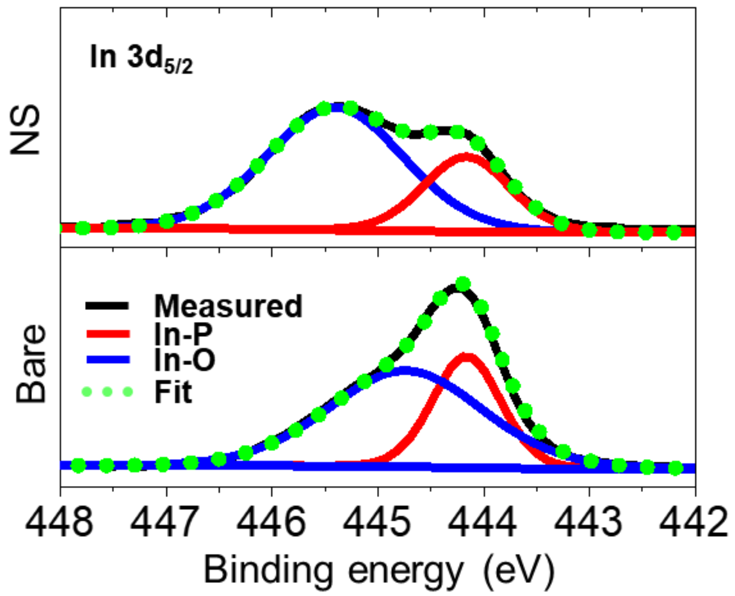

Supplementary Figure 8. XPS spectra of In $3d_{5/2}$

XPS spectra of In $3d_{5/2}$ for bare and nanostructured $GaInP_2$ (yet without $(NH_4)_2S$-treatment). The measured spectra (black line) matched quantitatively with the fitted spectra (green dotted line) composed of deconvoluted In-O (blue line) and In-P (red line) peaks.

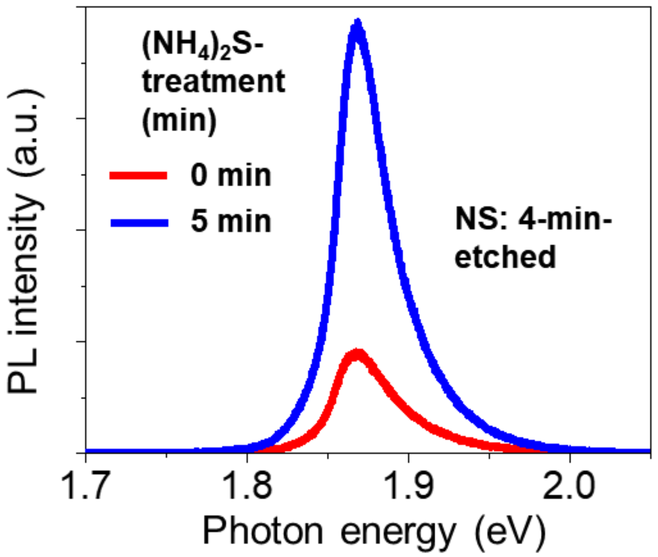

Supplementary Figure 9. Photoluminescence of $(NH_4)_2S$-treated black $GaInP_2$
Photoluminescence intensities of nanostructured (4-min-etched) $GaInP_2$ before and after $(NH_4)_2S$-treatment.

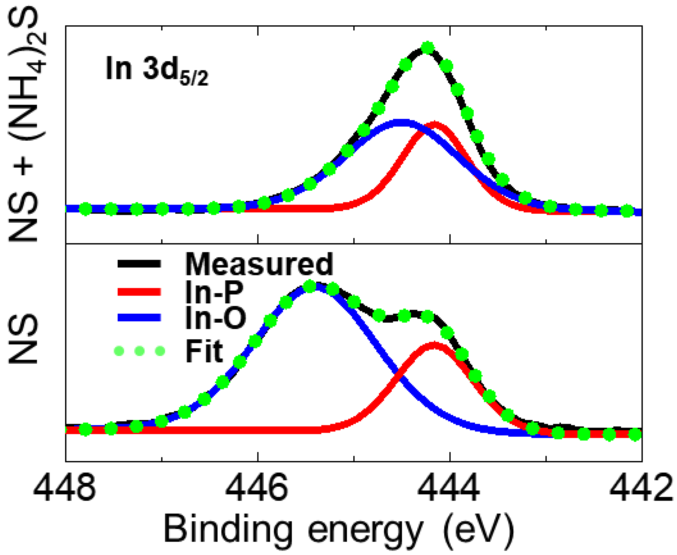

Supplementary Figure 10. XPS spectra of In $3d_{5/2}$

XPS spectra of In $3d_{5/2}$ for nanostructured $GaInP_2$ before and after the $(NH_4)_2S$-treatment. The measured spectra (black line) matched quantitatively with the fitted spectra (green dotted line) composed of deconvoluted In-O (blue line) and In-P (red line) peaks.

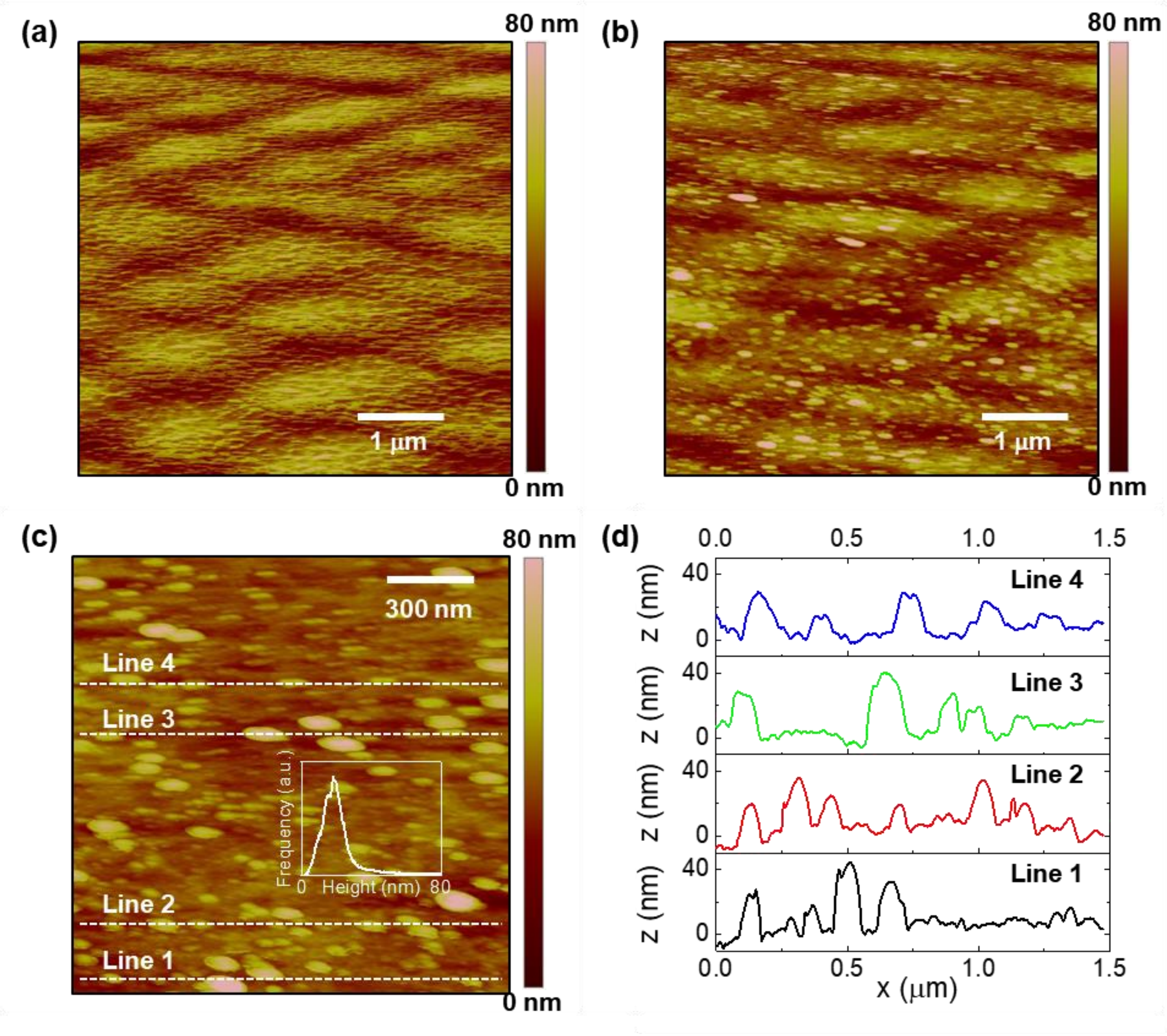

Supplementary Figure 11. AFM images of MoS_2 deposited on bare $GaInP_2$

Tapping-mode AFM images of (a) bare $GaInP_2$ and (b) MoS_2-deposited bare $GaInP_2$. (c) Zoomed-in image of MoS_2-deposited bare $GaInP_2$. The inset shows the distribution of height on the image. (d) The height (z) profiles corresponding to the scan lines in (c).

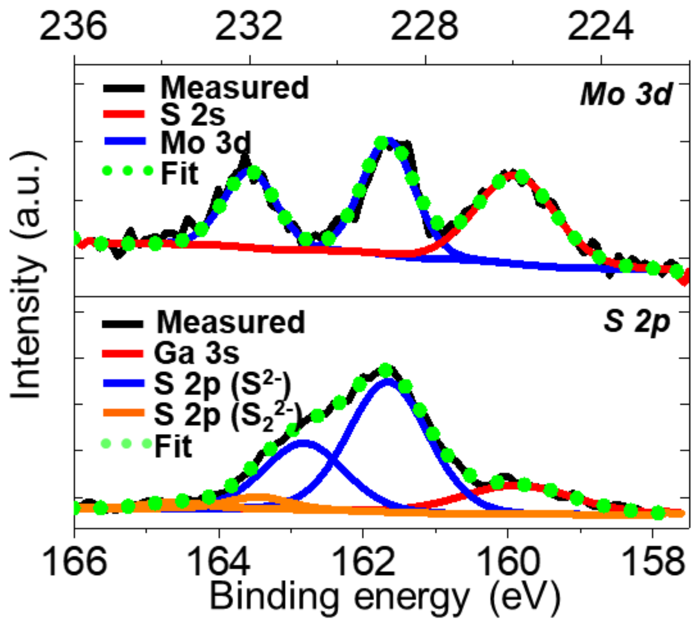

Supplementary Figure 12. XPS spectra of MoS_2

XPS spectra of Mo 3d and S 2p of amorphous molybdenum disulfide (MoS_2) deposited on $GaInP_2$ as a HER co-catalyst.

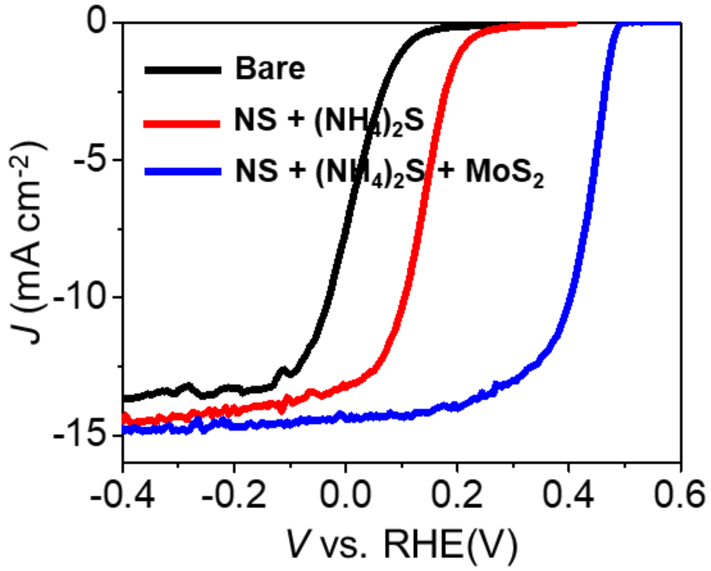

Supplementary Figure 13. PEC performance of sulfur-treated black $GaInP_2$
Representative *J-E* curves of bare $GaInP_2$ (black line), nanostructured $GaInP_2$ after $(NH_4)_2S$-treatment (red line), and nanostructured $GaInP_2$ after $(NH_4)_2S$-treatment and MoS_2 deposition (blue line), measured in an acidic electrolyte (0.5M H_2SO_4) under simulated AM 1.5G illumination.

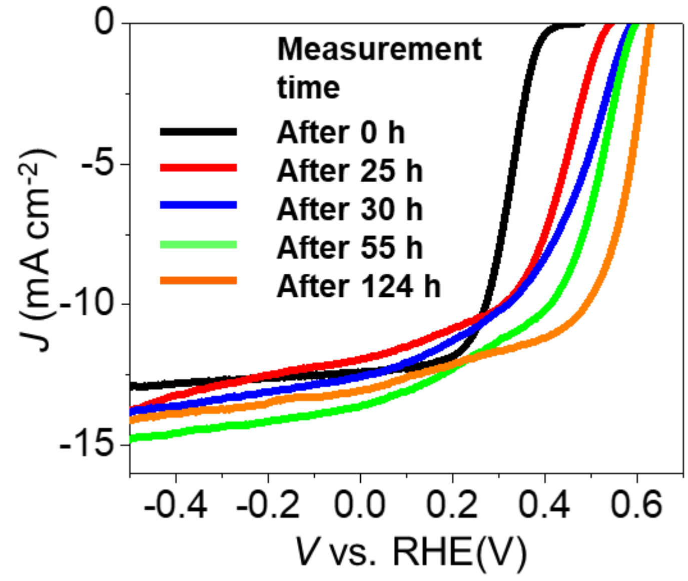

Supplementary Figure 14. PEC performance of sulfur-treated black $GaInP_2$
J-E curves of nanostructured and $(NH_4)_2S$-treated $GaInP_2$ measured during the stability test in Figure 4b.

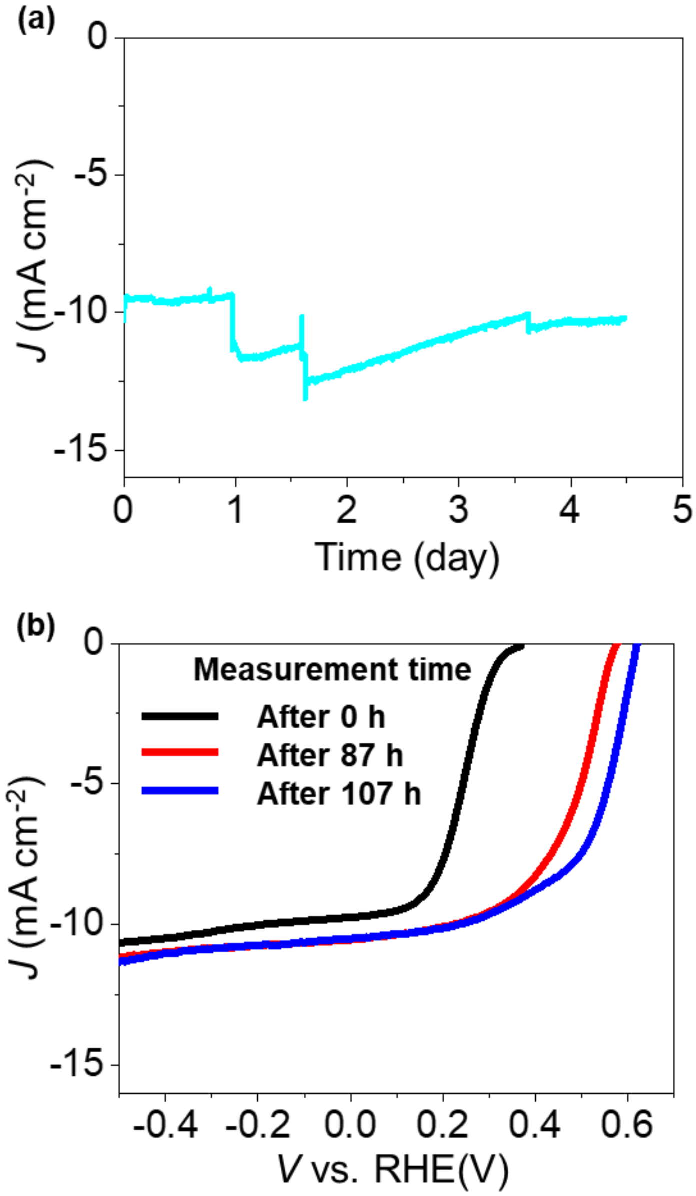

Supplementary Figure 15. Stability of sulfur-treated black $GaInP_2$

(a) Current density–time (*J*–*t*) plot obtained from another sample of nanostructured and $(NH_4)_2S$-treated $GaInP_2$ photocathode, measured under the same experimental condition as in Figure 4b. This semi-quantitative measurement confirms the extraordinary behavior of the electrode with both nanostructured morphology and $(NH_4)_2S$-treatment. (b) Corresponding *J-E* curves measured at the beginning as well as after 87 h and 107 h of the stability test.

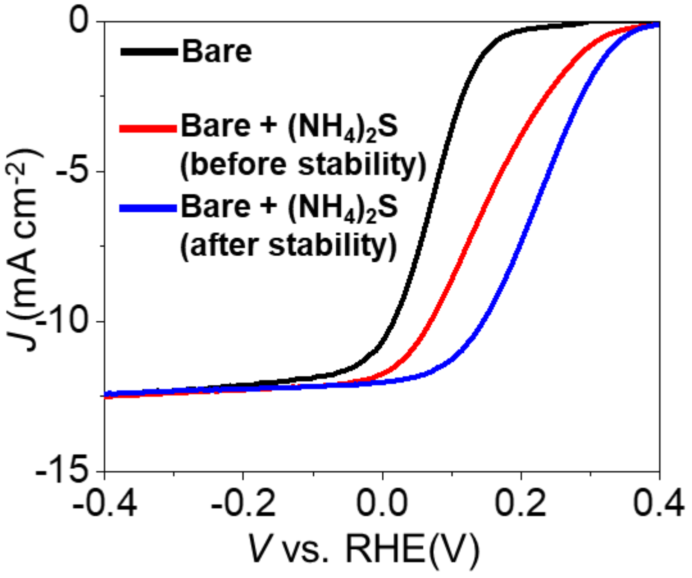

Supplementary Figure 16. PEC performance before and after the stability test
Representative *J-E* curves of $(NH_4)_2S$-treated bare $GaInP_2$ before (red) and after (blue) the 1-h stability test (Fig 4f), measured in an acidic electrolyte (0.5M H_2SO_4) under simulated AM 1.5G illumination. The *J-E* curve from the bare $GaInP_2$ (i.e. without $(NH_4)_2S$-treatment) is also shown for comparison.

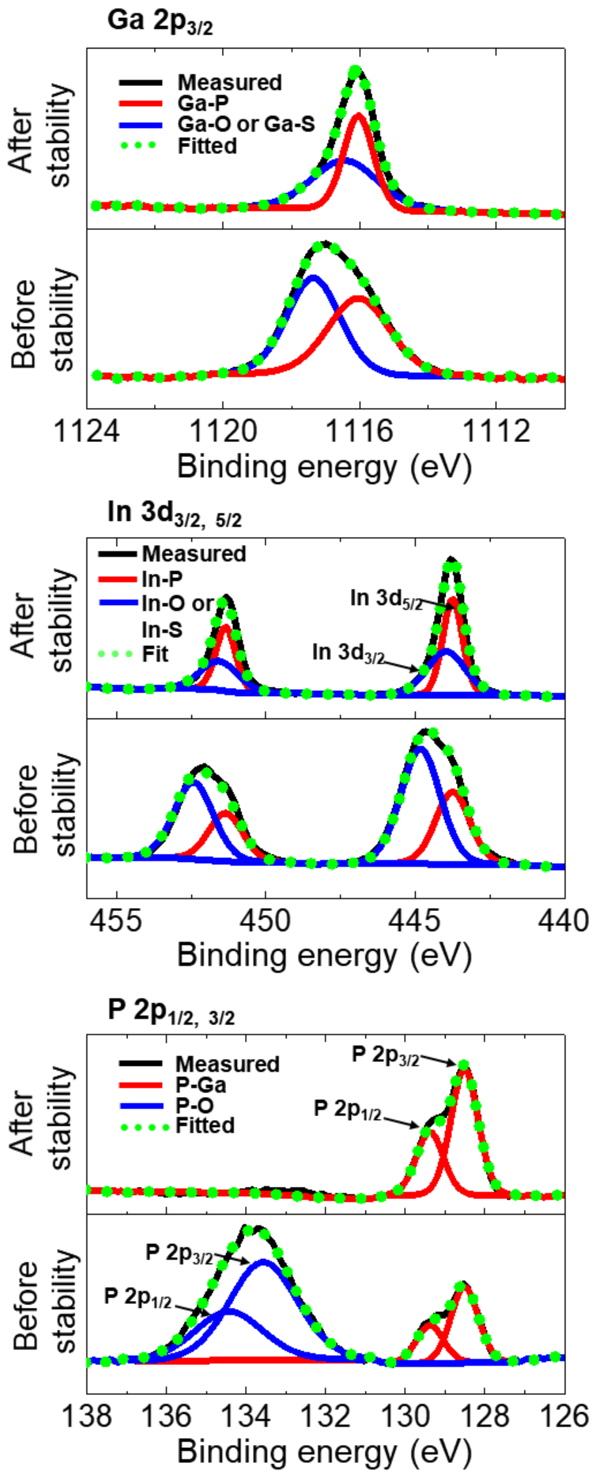

Supplementary Figure 17. XPS spectra before and after stability test

XPS spectra of Ga 2p, In 3d, and P 2p obtained from $(NH_4)_2S$-treatd bare $GaInP_2$ photocathode before and after the stability test performed for 1 h shown in Fig. 4f.

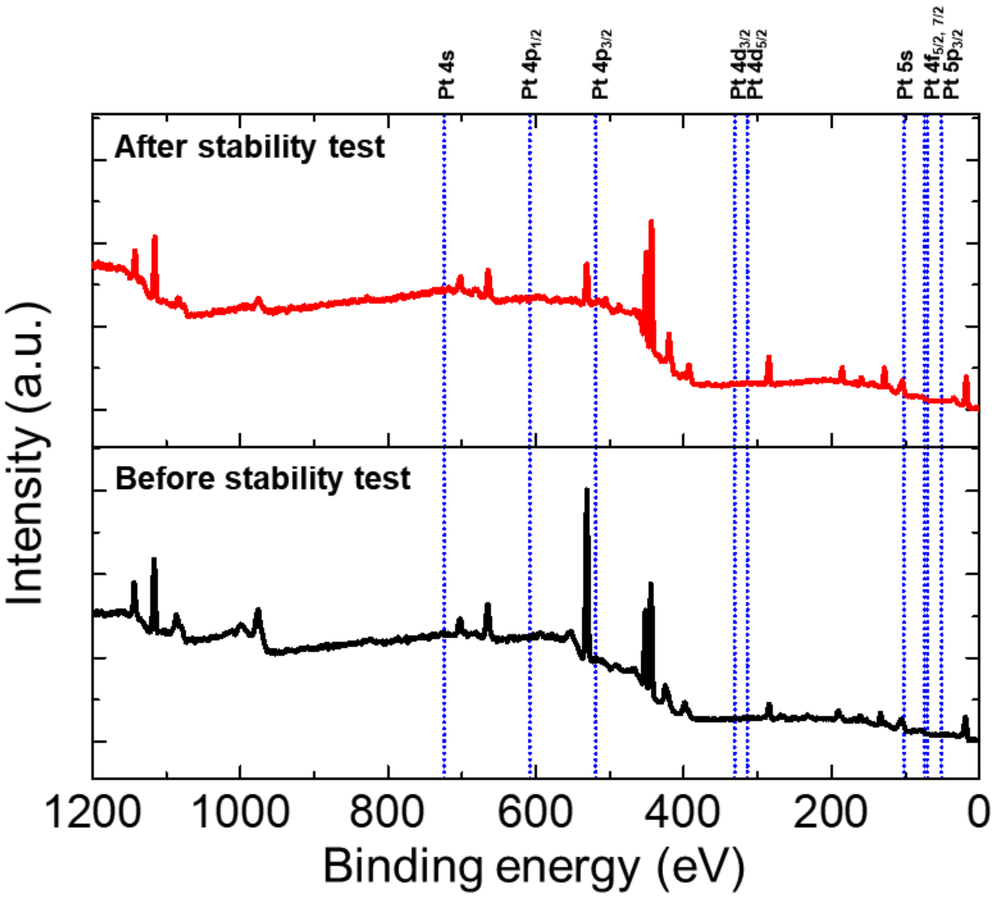

Supplementary Figure 18. XPS spectra for Pt

Pt-related XPS survey spectra of $(NH_4)_2S$-treated bare $GaInP_2$ photocathode before and after the stability test (Fig. 4f).

(a)

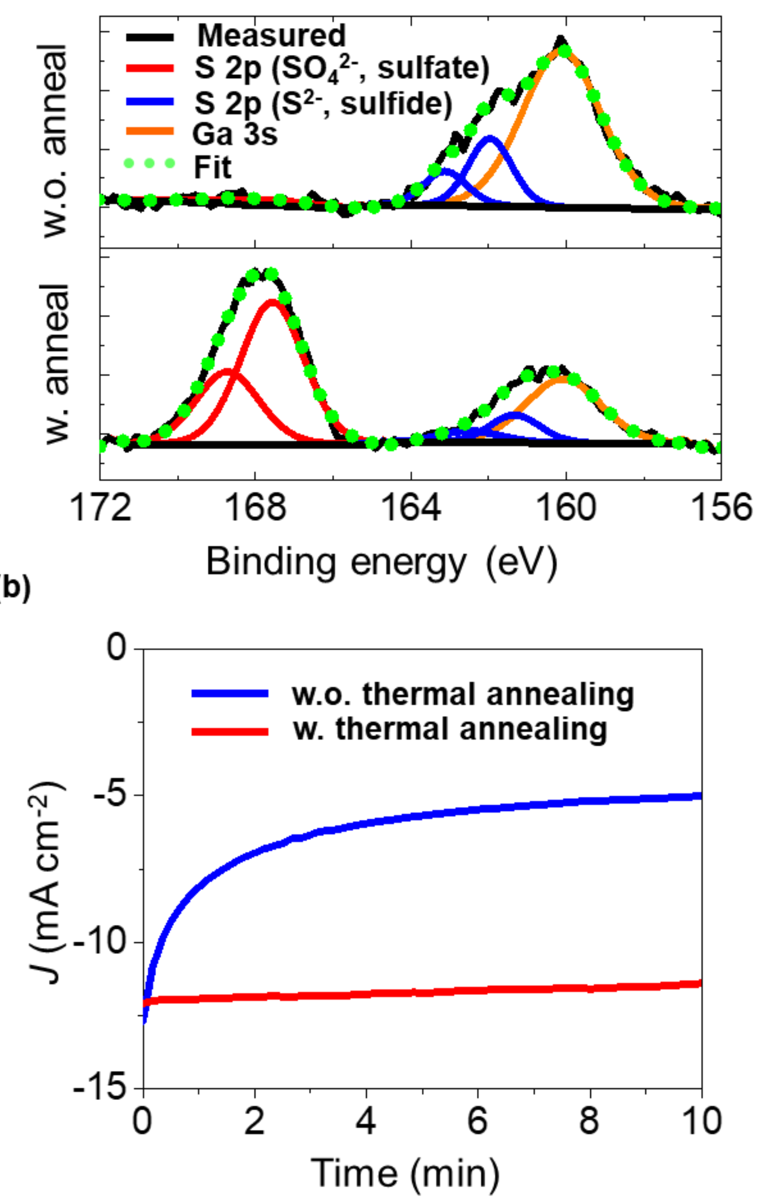

Supplementary Figure 19. Effect of thermal annealing

(a) XPS spectra of S 2p and Ga 3s measured from bare $GaInP_2$ photocathodes with and without thermal annealing (250°C, 1 h, in air) during the $(NH_4)_2S$-treatment. (b) Representative *J-E* curves of bare $GaInP_2$ photocathodes with and without thermal annealing during the $(NH_4)_2S$-treatment, measured in an acidic electrolyte (0.5M H_2SO_4) under simulated AM 1.5G illumination.

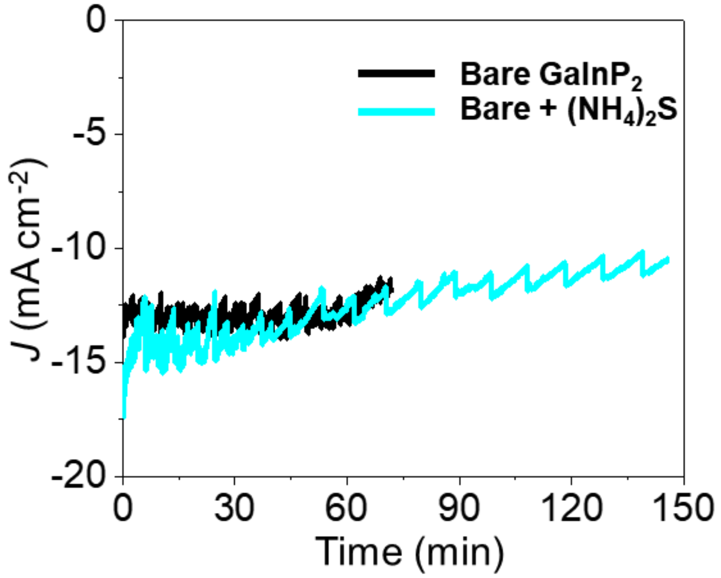

Supplementary Figure 20. ***J-t*** **plots during FE measurements**
J-t plots of bare $GaInP_2$ photocathodes with and without $(NH_4)_2S$-treatment, obtained from Faradaic efficiency measurements shown in Supplementary Table 5.

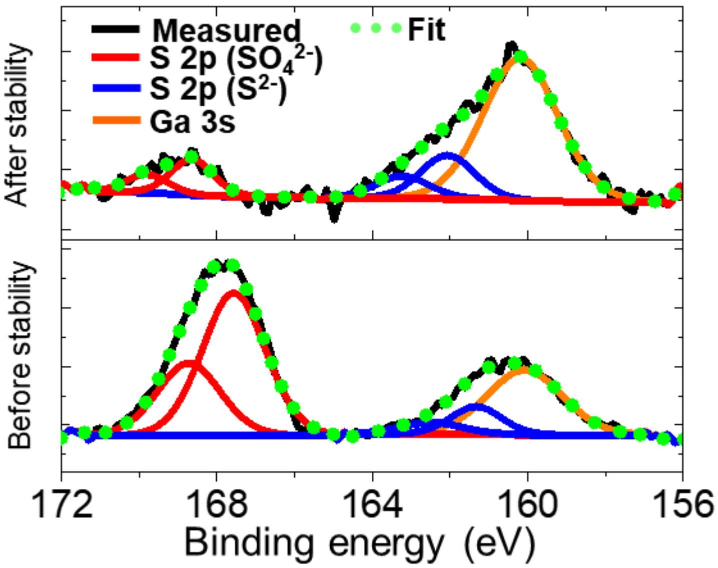

Supplementary Figure 21. XPS spectra before and after stability test
XPS spectra of S 2p and Ga 3s from bare $GaInP_2$ photocathodes with $(NH_4)_2S$-treatment before and after the stability test (*J*-*t*) for 1 h shown in Figure 4f (cyan data). After the stability test, the relative area of sulfate-related peaks decreased.

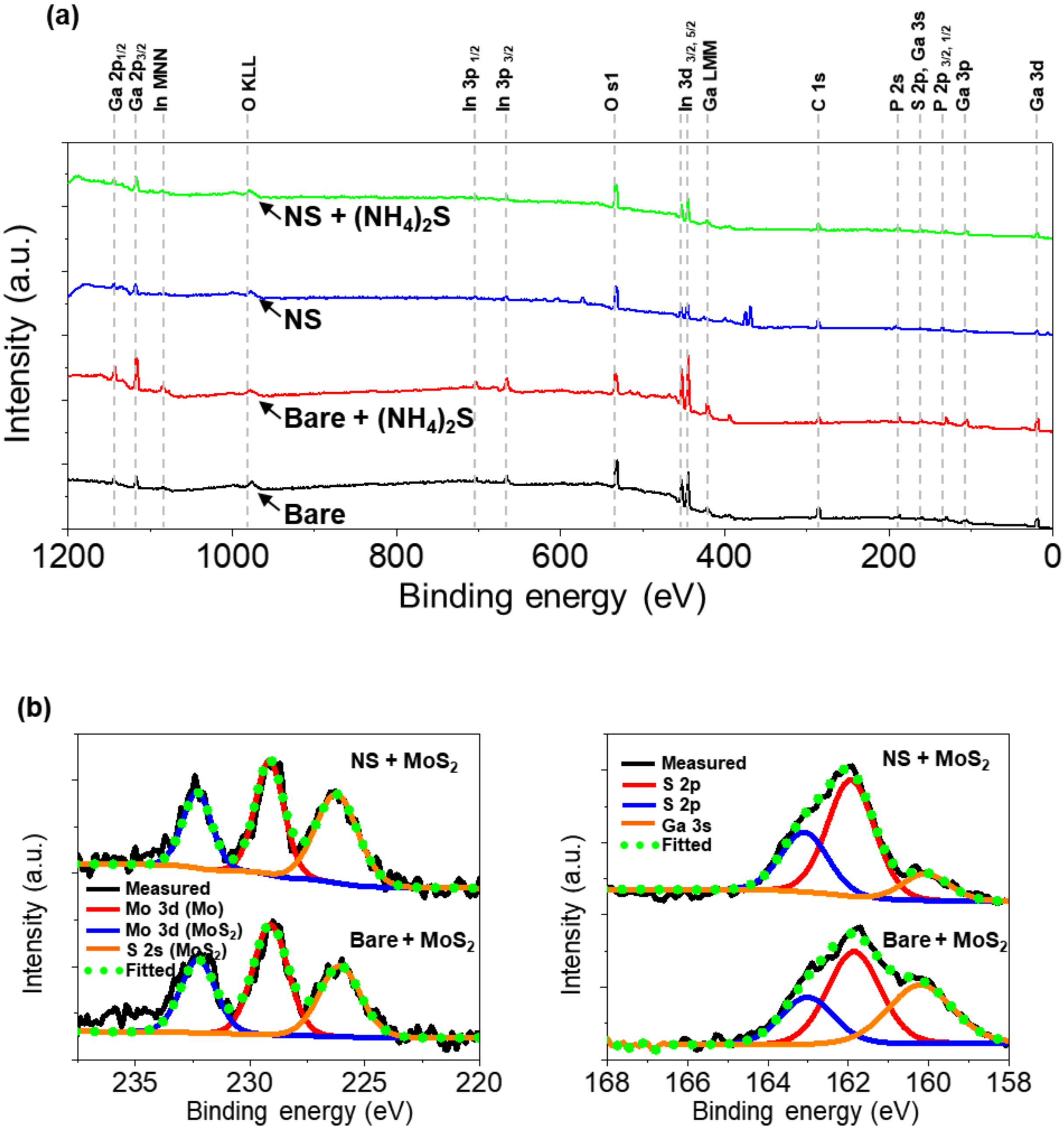

Supplementary Figure 22. XPS spectra after $(NH_4)_2S$-treatment and MoS_2 deposition
(a) XPS survey spectra of bare and nanostructured $GaInP_2$ photocathode before and after $(NH_4)_2S$-treatment (b) XPS spectra of Mo 3d and S p2 from bare (lower) and nanostructured (upper) $GaInP_2$ after MoS_2-deposition, showing that the peak positions and relative intensity ratios are similar between bare and nanostructured samples.

Supplementary Tables

Etch time (min)	J_{sat}[a] (mA cm^{-2})	V_{onset}[b] (V)	*FF*	$\eta_{cathode}$ (%)
**0**	-12.7	0.155	0.195	0.29
**1**	-13.5	0.284	0.230	0.79
**2**	-6.0	0.314	0.540	0.98
**3**	-8.0	0.284	0.442	0.90
**4**	-9.0	0.309	0.412	1.07

[a]J_{sat} (saturation current density) was determined at the voltage of -0.5 V vs. RHE
[b]V_{onset} (onset potential) was decided at the current density of -0.5 mA cm^{-2}

Supplementary Table 1. Photoelectrochemical (PEC) performance characteristics of nanostructured $GaInP_2$ photocathodes performing the HER, extracted from Figure 2a.

	J_{sat} (mA cm^{-2})	V_{onset} (V)	*FF*	$\eta_{cathode}$ (%)
**Bare, 0 min[b]**	-13.3	0.105	0.178	0.11
**NS[a], 0 min**	-10.9	0.275	0.362	1.02
**NS, 3 min**	-14.4	0.314	0.294	1.25
**NS, 5 min**	-15.2	0.271	0.360	1.40

[a]4-min etched $GaInP_2$
[b]time of $(NH_4)_2S$-treatment

Supplementary Table 2. Photoelectrochemical (PEC) performance characteristics of bare and nanostructured $GaInP_2$ Photocathodes performing the HER, extracted from Figure 3a.

	J_{sat} (mA cm^{-2})	V_{onset} (V)	*FF*	$\eta_{cathode}$ (%)
**Bare**	-13.6	0.123	0.195	0.20
**NS[a] + $(NH_4)_2S$[b]**	-14.7	0.240	0.329	1.05
**NS + $(NH_4)_2S$ + MoS_2**	-14.9	0.483	0.621	4.32

[a] 1-min etched $GaInP_2$
[b] 10-min $(NH_4)_2S$-treatment

Supplementary Table 3. Photoelectrochemical (PEC) performance characteristics of nanostructured $GaInP_2$ photocathodes deposited with MoS_2 performing the HER, extracted from Figure S10.

Year	Materials	Rxn[a]	Protection layer	Stability[b]	Electrolyte	$J_{ini}{}^{c}$	$J_{fin}{}^{d}$	$\%_{red}{}^{e}$	Ref.
2018	p-$GaInP_2$	HER	**None**	**124 h**	0.5M H_2SO_4	-12.7	-12.8	**-1%**	**This work**
2017	p-$GaInP_2$	HER	TiO_2/g-MoS_x	20 h	0.5M H_2SO_4	-11.2	-9.2	17%	***Nature Energy*** 2, 16192, (2017)
2017	n-$GaInP_2$ (GaInP/ GaInAs)	HER	PtRu	12 h	3M H_2SO_4	-14.0	-11.3	20%	***Nature Energy*** 2, 17028, (2017)
2016	p-$GaInP_2$	HER	Mo/MoS_2	70 h	3M H_2SO_4	-6.7	-5.8	14%	***Journal of Physical Chemistry Letters*** 7, 2044, (2016).
2016	n^+p-InP	HER	TiO_2/Pt	6 h	1M $HClO_4$	-27.0	-26.7	1%	***Advanced Functional Materials*** 26, 679, (2016).
2016	p-$GaInP_2$	HER	TiO_2/Co-TiO_2	20 min	0.5M NaOH	-9.1	-7.57	17%	***Nature Materials*** 15, 456, (2016).
2014	p^+n-GaAs	OER	TiO_2/Ni	26 h	1M KOH	-14.0	-12.7	9%	***Science*** 344, 1005, (2014).
2014	p-GaP	HER	TiO_2/Pt	24 h	1M $HClO_4$	-1.0	-0.9	10%	***Journal of Materials Chemistry A*** 2, 6847, (2014).

[a]Electrochemical reaction (HER: hydrogen evolution reaction, OER: oxygen evolution reaction)
[b]Duration between the beginning and end of the stability test, all performed at 0 V vs. RHE.
[c]Initial current density (mA cm^{-2}) at the beginning of the stability test
[d]Final current density (mA cm^{-2}) at the end of the stability test
[e]Percentage of reduction in the current density of photoelectrodes, calculated by $(J_{ini}-J_{fin})/J_{ini} \times 100$

Supplementary Table 4. Summary of published stability data of III-V compound semiconductor photoelectrodes in solar water splitting.

	Gas	Time (sec)	Averaged current (mA)	Total charge passed (C)	Expected gas quantity (mol)	Measured gas volume (mL)	h_1 (mm)	P_{H2} or P_{O2} (torr)	Calculated gas quantity (mol)	Faradaic efficiency
Pt	H_2	600	10	6.00	3.11E-05	0.94	30	596.0	3.07E-05	0.99
Pt	O_2	600	10	6.00	1.55E-05	0.47	5	597.9	1.54E-05	0.99
$(NH_4)_2S$-treated bare $GaInP_2$	H_2	8740.2	0.845	7.39	3.83E-05	1.06	26	596.3	3.46E-05	0.91
Pt	O_2	8740.2	0.845	7.39	1.91E-05	0.59	0	598.2	1.93E-05	1.01
Untreated bare $GaInP_2$	H_2	4311	1.872	8.07	4.18E-05	1.02	27	596.3	3.33E-05	0.80
Pt	O_2	4311	1.872	8.07	2.09E-05	0.66	0	598.2	2.16E-05	1.03

Supplementary Table 5. Values for determining faradaic efficiency of bare $GaInP_2$ photocathodes with and without $(NH_4)_2S$-treatment.

Supplementary Notes

Supplementary Note 1. Improvement of onset potential during the stability measurements of $(NH_4)_2S$-treated $GaInP_2$ photocathodes

We attribute the improvement of onset potential for $(NH_4)_2S$-treated $GaInP_2$ during the stability test (Supplementary Figs. 14-16) to several factors. One possible reason is associated with the gradual activation of catalytic sites of sulfurized layers of $GaInP_2$ with the dissolution of oxide and carbon-containing species that were formed during the electrode preparation and are unstable in sulfuric acid under the electrochemical condition of HER. We have consistently observed the anodic shift of onset potential from $(NH_4)_2S$-treated bare and nanostructured $GaInP_2$ photocathodes during the HER compared to the case without $(NH_4)_2S$-treatment, supporting the catalytic effect of the sulfurized surface of $GaInP_2$. This postulate is also consistent with the XPS spectra (Supplementary Fig. 17) of Ga, In, and P obtained from $(NH_4)_2S$-treated bare $GaInP_2$ before and after the stability measurements, where oxide-related XPS signals all considerably decreased after the stability measurements. Secondly, such removal of surface oxides can also lead to the reduction of surface states for carrier recombination[1,2] and thus improve the photovoltage of $GaInP_2$, which in turn can shift the onset potential anodically. Thirdly, carbon-containing species that were formed after the $(NH_4)_2S$-treatment (as shown in the EDS analysis of Supplementary Fig. 1) might be removed in sulfuric acid to improve the efficiency of charge transfer and thus reduce the chemical overpotential, thereby resulting in the anodic shift of onset potential. Fourth, sulfur may also act as n-type dopant[3,4] to form a buried junction and increase the photovoltage.

Supplementary Note 2. Area of tested photoelectrodes

Current density (*J*) in all *J*-*E* plots was evaluated using the measured area of illuminated electrode surface. The electrode area of samples was determined from individual photographic images of samples by an image analysis software (e.g. Adobe®Photoshop®). Below is the list of sample area used to evaluate current density in Figs. 2a, 3a, 4a, 4b, and 4f.

■ **Figure 2a**

0 min (bare) (black line) : 0.1144 cm^2
1 min (red line) : 0.0482 cm^2
2 min (blue line) 0.0427 cm^2
3 min (green line) 0.0522 cm^2
4 min (orange line) : 0.0286 cm^2

■ **Figure 3a**

0 min (bare) (black line) : 0.6541 cm^2
0 min (NS) (red line) : 0.0643 cm^2
3 min (NS) (blue line) : 0.1315 cm^2
5 min (NS) (green line) : 0.0405 cm^2

■ **Figure 4a**

Bare (black line) : 0.1534 cm^2

Bare + MoS_2 (red line) : 0.2599 cm^2
NS + $(NH_4)_2S$ (blue line) : 0.0405 cm^2
NS + MoS_2 (green line) : 0.0432 cm^2

- **Figure 4b**
  Bare (black line) : 0.1534 cm^2
  Bare + MoS_2 (red line) : 0.2599 cm^2
  NS + $(NH_4)_2S$ (blue line) : 0.0405 cm^2
  NS + MoS_2 (green line) : 0.0432 cm^2

- **Figure 4f**
  Bare (black line) : 0.1846 cm^2
  Bare + $(NH_4)_2S$ (cyan line) : 0.0977 cm^2
  NS (orange line) : 0.0360 cm^2
  NS + $(NH_4)_2S$ (blue line) : 0.0405 cm^2

Supplementary References

1. Young, J. L., Döscher, H., Turner, J. A. & Deutsch, T. G. Reversible GaInP2 Surface Passivation by Water Adsorption: A Model System for Ambient-Dependent Photoluminescence. The Journal of Physical Chemistry C 120, 4418-4422, (2016).

2. Dean, P. J., Henry, C. H. & Frosch, C. J. Infrared Donor-Acceptor Pair Spectra Involving the Deep Oxygen Donor in Gallium Phosphide. Physical Review 168, 812-816, (1968).

3. Bove, P., Garcia, J. C., Maurel, P. & Hirtz, J. P. Sulfur doping of GaAs and GaInP grown by metalorganic molecular beam epitaxy using a hydrogen sulfide gaseous source. Applied Physics Letters 58, 1973-1975, (1991).

4. Lee, J.-L. Sulfur doping of GaAs with (NH4)2Sx solution. Journal of Applied Physics 85, 807-811, (1999).
